# Long-Term Outcomes of Multimodal Prehabilitation with High Protein Oral and HMB Supplementation in Sarcopenic Surgical Patients: The HEROS Study

**DOI:** 10.3390/nu18040703

**Published:** 2026-02-22

**Authors:** Irving Yu Le Shua, Yong Yi Tan, Vanessa Yik, Jing Han Hong, Yun-Xia Liu, Shuen-Ern Chin, Shawn Shi-Xian Kok, Hui-Bing Lee, Cherie Tong, Phoebe Tay, Esther Chean, Yi-En Lam, Shi-Min Mah, Li-Xin Foo, Clement C. Yan, Wei-Tian Chua, Haziq bin Jamil, Khasthuri Ganesh, Lester Wei-Lin Ong, Alvin Yong-Hui Tan, Koy-Min Chue, Leonard Ming-Li Ho, Cheryl Xi-Zi Chong, Jasmine Ladlad, Cheryl Hui-Min Tan, Nathanelle Ann Xiaolian Khoo, Jia-Lin Ng, Winson Jianhong Tan, Fung-Joon Foo, Bin Tean Teh, Yibin Wang, Frederick Hong-Xiang Koh

**Affiliations:** 1 Lee Kong Chian School of Medicine, Nanyang Technological University, Singapore 308232, Singapore; shua0002@e.ntu.edu.sg (I.Y.L.S.); m220087@e.ntu.edu.sg (Y.Y.T.); chin.shuen.ern@mohh.com.sg (S.-E.C.); 2 Duke-NUS Medical School, National University of Singapore, Singapore 169857, Singapore; vanessa.yik.yijean@mohh.com.sg (V.Y.); jinghan.hong@duke-nus.edu.sg (J.H.H.); yunxia.liu@duke-nus.edu.sg (Y.-X.L.); yibinwang@duke-nus.edu.sg (Y.W.); 3 SingHealth Duke-NUS Muscle Health Programme, Singapore 168582, Singapore; teh.bin.tean@singhealth.com.sg; 4 Department of Radiology, Sengkang General Hospital, Singapore 544886, Singapore; drshawnkok@gmail.com (S.S.-X.K.); esther.chean.y.r@skh.com.sg (E.C.); lam.yi.en@skh.com.sg (Y.-E.L.); 5 Department of Dietetics, Sengkang General Hospital, Singapore 544886, Singapore; lee.hui.bing@skh.com.sg (H.-B.L.); cherie.tong.c.y@skh.com.sg (C.T.); phoebe.tay.d.w@skh.com.sg (P.T.); 6 Department of Physiotherapy, Sengkang General Hospital, Singapore 544886, Singapore; mah.shi.min@skh.com.sg (S.-M.M.); foo.li.xin@skh.com.sg (L.-X.F.); clement.yan.c.k@skh.com.sg (C.C.Y.); 7 Department of Colorectal Surgery, Sengkang General Hospital, Singapore 544886, Singapore; chua.wei.tian@skh.com.sg (W.-T.C.); muhammad.haziq.jamil@skh.com.sg (H.b.J.); g.khasthuri@skh.com.sg (K.G.); leonard.ho.m.l@singhealth.com.sg (L.M.-L.H.); cheryl.chong.x.z@singhealth.com.sg (C.X.-Z.C.); ladlad.jasmine.guintu@skh.com.sg (J.L.); cheryl.tan.h.m@skh.com.sg (C.H.-M.T.); nathanelle.ann.k.x@singhealth.com.sg (N.A.X.K.); ng.jia.lin@singhealth.com.sg (J.-L.N.); winson.tan.j.h@singhealth.com.sg (W.J.T.); foo.fung.joon@singhealth.com.sg (F.-J.F.); 8 Department of General Surgery, Sengkang General Hospital, Singapore 544886, Singapore; lester.ong.w.l@singhealth.com.sg (L.W.-L.O.); alvin.tan.y.h@singhealth.com.sg (A.Y.-H.T.); chue.koy.min@singhealth.com.sg (K.-M.C.)

**Keywords:** prehabilitation, sarcopenia, oral nutritional beta-hydroxy beta-methylbutyrate, branched chain amino acid, intramuscular adipose tissue

## Abstract

**Background:** Sarcopenia is associated with adverse surgical outcomes. Recent literature suggested that pre-surgery prehabilitation, nutrition and β-Hydroxy β-methylbutyric acid (HMB) supplementation improve myological and functional outcomes. However, long-term outcomes remain uncertain. Thus, we aimed to analyse findings from the long-term follow-up of surgical patients undergoing oral supplementation and prehabilitation. **Methods:** A prospective single-centre pilot cohort study was conducted to evaluate the effects of multimodal prehabilitation using high-protein oral nutritional supplementation (HP-ONS) with HMB. Sarcopenic patients between 40 and 90 years old and undergoing gastrointestinal surgery were included from June 2022 to January 2024. Patients were followed up from two to four weeks pre-operatively to six months post-operatively. Paired Wilcoxon signed-rank tests were conducted to evaluate outcomes between time points. **Results:** 36 patients were included with a median age of 71.5 years (IQR: 55–90), and 50% were male. 12 (33.3%) patients were sarcopenic, while 24 (66.7%) were severely sarcopenic. No significant difference in IMAT% was observed at post-operative six months. Number of chair rise repetitions (Median:15 vs. 11, *p* < 0.001) and six-min walk test (Median: 387 m vs. 349 m, *p* = 0.020), mid-arm muscle circumference (Median: 23.7 cm vs. 22.5 cm, *p* = 0.013), mid-arm muscle area (Median: 44.8 cm^2^ vs. 39.3 cm^2^, *p* = 0.005), weight (Median: 51.6 kg vs. 50.8 kg, *p* = 0.023) and BMI (Median: 23.2 kg/m^2^ vs. 21.8 kg/m^2^, *p* = 0.025) increased significantly at six-months post-operatively from surgery. **Conclusions:** Our findings suggest that improvements in anthropometric and functional outcomes from prehabilitation may persist beyond the acute recovery period. If supported by future works, multimodal prehabilitation has the potential to optimise postoperative recovery amongst sarcopenic older adult surgical patients.

## 1. Introduction

### 1.1. Background

Sarcopenia, the progressive loss of muscle mass and function, is often associated with adverse outcomes [1]. Globally, an estimated 16% of the older adult population has sarcopenia, and the prevalence is even higher amongst patients [2]. In Singapore, 20% of older adults aged 65 and above have low appendicular skeletal muscle mass index [3]. Another study showed that 88% of older adults in Singapore had poor muscle health, with 44% having sarcopenia [4]. Multiple studies have demonstrated that sarcopenic patients undergoing surgery often have greater surgical complications, post-operative mortality and overall increased cost [5,6], be it for gastrointestinal surgery [7], hepatic surgery [8], orthopaedic surgery [9] or oncologic surgery [10]. Thereby, prehabilitation before surgery, to combat sarcopenia, has been shown to improve outcomes in these patients [6,11,12].

### 1.2. Previous Literature

The concept of a multimodal prehabilitation regime has become more prominent, suggesting that an effective prehabilitation programme should comprise nutritional, psychological and physical components [6,11,13,14]. Preoperative optimisation through prehabilitation is thought to increase metabolic and physiological reserves of the patient in preparation for an acute stressor such as surgery [14,15,16]. Physical exercise during prehabilitation has been reported to decrease post-operative mortality rates and complications in abdominal [17,18] and gastric surgery [19]. High protein oral nutritional supplementation (HP ONS) has been shown to improve functional exercise capacity and decrease length of stay in gastrointestinal surgery [19,20,21]. The evidence suggests that, together in a multimodal prehabilitation programme, there is a more holistic recovery, including clinical and functional benefits in patients undergoing surgery [20]. Additionally, beta-hydroxy beta-methylbutyrate (HMBs) supplementation, a metabolite of the essential branched-chain amino acid (BCAA) leucine, has been reported to preserve and even potentially improve muscle mass and function in older adults and sarcopenic populations [22,23,24]. Proposed mechanisms by which HMB might improve muscle mass and quality include inhibiting muscle proteolysis during stress and exercise by enhancing myocyte membrane integrity [25,26], as well as inducing hypertrophy by stimulating muscle stem cell proliferation [27]. The use of HMB during prehabilitation is relatively novel but has gained traction, being trialled in orthopaedic [12,28] and cardiac [29] surgery. Although there remain other nutritional supplements for muscle recovery, such as vitamin D, creatine and protein supplementation, such supplements often either enhance performance and function, or promote anabolic pathways [30]. In surgical patients with or at risk of sarcopenia, accelerated muscle catabolism is a significant driver of muscle loss and loss-of-function rather than suboptimal muscle anabolism. As such, HMB has potential for more advantageous outcomes in surgical patients due to its hypothesised inhibition of muscle proteolysis. Furthermore, HMB, a metabolite of leucine, is not readily available endogenously, as less than 1% of leucine turnover is accounted for by HMB [31]. As such, HMB supplementation may be the most practical and feasible approach towards achieving clinical intake targets of 3 g/day, which would otherwise be difficult to achieve through diet alone, such that potential inhibition of muscle proteolysis might be observed.

### 1.3. Current Gaps

In our earlier publication of preliminary results from the Oral Nutritional Supplementation with HMB enhanced muscle quality in sarcopenic surgical patients (HEROS) study, we found that the prehabilitation regime was associated with an improvement in gait speed after 2 weeks of prehabilitation, which subsequently returned to baseline post-operatively [32]. There was also an association with an increase in IMAT index, sustained up to 1 month post-operatively [32]. However, the long-term benefits of perioperative optimisation through a multimodal prehabilitation have been understudied, with most studies usually reporting outcomes up to 30 days [33,34]. The numbers are fewer when relating specifically to sarcopenic patients undergoing gastrointestinal surgery. It remains unclear whether the changes in muscle quality and functional outcomes post-prehabilitation are sustained post-operatively by these patients, especially after ceasing the intervention [35]. The benefits of resistance exercise and nutritional supplementation in the management and prevention of sarcopenia have been clearly delineated [36,37,38,39,40]. Coincidentally, these are also cornerstones in prehabilitation may experience long-term behavioural changes and health benefits after exposure to the multidimensional intervention [35,41]. Together, the preservation of these habits could potentially be used to combat the pre-existing sarcopenia in these patients, leading to an overall improvement in the physiological and functional outcomes of these patients in the long run [42,43].

### 1.4. Aims and Objectives

Previously, we have shown the feasibility of the prehabilitation regime and the short-term functional benefits of such a regime [32]. In this extended analysis, we aim to assess the long-term importance of a multimodal prehabilitation programme incorporating HP ONS with HMB and resistance exercise on the muscle quality, functional outcomes, and quality of life of sarcopenic surgical patients up to 6 months after undergoing gastrointestinal surgery. We hypothesize that improvements in muscle quality in terms of IMAT measures, functional outcomes such as grip strength and 6-min-walk test (6MWT) and quality-of-life measures are sustained following multimodal prehabilitation in a cohort of surgical patients with sarcopenia.

## 2. Materials and Methods

### 2.1. Study Design

A prospective non-randomised interventional pilot cohort study was performed in a single institution to evaluate the effect of a multimodal prehabilitation program using an HP ONS with HMB and resistance exercise in sarcopenic patients undergoing surgery. This is an extended outcome analysis of the study protocol titled “Oral Nutritional Supplementation with HMB enhanced muscle quality in sarcopenic surgical patients (HEROS)—a pilot interventional cohort study.” [32]. This study was approved by the SingHealth Centralized Institute Review Board (CIRB: 2022/2027) and registered with ClinicalTrials.gov (NCT05344313) on 25 April 2022.

Patients included in this study were adult patients aged between 40 and 90 years, sarcopenic according to the Asian Workgroup of Sarcopenia (AWGS) 2019 diagnostic criteria, scheduled to undergo elective major gastrointestinal surgery, ambulant, and able to comply with physiotherapy and dietitian advice. Patients who fulfilled the Asian Working Group for Cachexia (AWGC) criteria for cachexia were excluded from recruitment [44]. Patients who were pregnant, had chronic kidney disease or had diabetes mellitus were excluded. Eligible patients were recruited from Sengkang General Hospital from June 2022 to January 2024, and written informed consent was obtained. The patients were evaluated for sarcopenia based on the Asian Working Group for Sarcopenia 2019 guidelines (AWGS 2019) with bioelectrical impedance analysis to assess appendicular skeletal mass [45].

**Figure 1:**
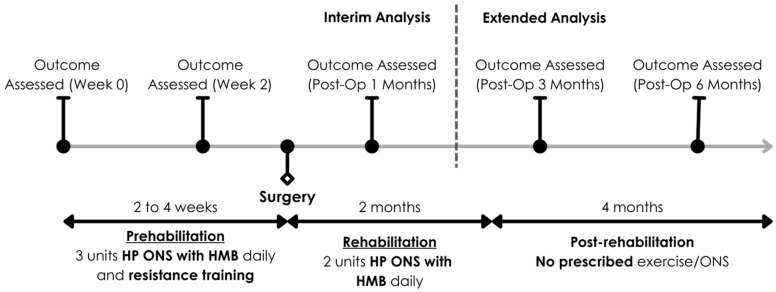
Diagram of patients’ journey through the HEROS study.

The multimodal prehabilitation regime in our study consisted of oral nutritional supplementation, resistance training exercises, vitamin D and iron supplementation, comorbidity optimization and smoking cessation. The patients were followed up after the initial 2–4 weeks of multimodal prehabilitation and a 4-week post-operative rehabilitation regimen [32]. Figure 1 illustrates the timeline of patient progression through the various stages of the HEROS study and components of the various phases. During the prehabilitation phase, 3 units of Ensure^®^ Plus Advance with HMB were prescribed daily. Individualised resistance training of upper and lower limbs was also prescribed with a regular once-weekly review. Post-operatively, patients were then prescribed 2 units (440 mL/day) of Ensure^®^ Plus Advance with HMB (Abbott Nutrition, Chicago, IL, USA) to consume daily for two months after surgery. Further details of the intervention of this study were published in the earlier interim analysis [32].

### 2.2. Outcomes Assessed

The equipment used, outcome variables and assessment methods were consistent with those from the interim analysis. Muscle quality, in terms of intramuscular adipose tissue (IMAT%) [46], IMAT index (ratio of IMAT% to muscle area), surface area of the rectus femoris (RFSA) and thickness of the rectus femoris (RFT) were assessed through ultrasound of the rectus femoris of both thighs, and the average was reported. IMAT% and IMAT index were derived using an Artificial Intelligence (AI)-aided program, MuscleSound^®^ (Denver, CO, USA). The midpoint of the rectus femoris was scanned, using standardized landmarks provided by MuscleSound^®^. Functional outcomes, including handgrip strength, 30 s chair rise, functional reach test, 6MWT and gait speed, and anthropometric measurements (mid-arm circumference, mid-arm muscle circumference, mid-arm muscle area and triceps skinfold) were also obtained. In addition, the nutritional status of the patients was assessed using the 7-point Subjective Global Assessment (SGA) by trained dietitians [47]. Patients’ subjective quality of life was assessed using the self-reported EuroQol-5 Dimension-3 Level (EQ5D-3L) questionnaire at baseline, post-operatively, 3 months and 6 months [48,49]. All other study outcomes (muscle quality, functional outcomes and anthropometric measurements) were again at three months after surgery (“Post-Op 3 Months”) and six months after surgery (“Post-Op 6 Months”). Further details on the methods of the abovementioned assessments can be found in the previously published article [32].

### 2.3. Statistical Analyses

All statistical analyses were performed using R Version 4.4.2 (R Core Team, 2025). Continuous variables were presented as median with range unless stated otherwise. Categorical variables were presented as numbers and percentages (%). Box and whisker plots were plotted to visualise the changes in muscle quality and functional outcomes over the perioperative period. Outliers were defined as any value that was two standard deviations greater than or below the mean. Given the small sample size, no outliers were excluded in the main analysis. However, sensitivity analyses excluding outliers were conducted to ensure that findings were not significantly driven by outlier values. Univariate analyses were conducted using the paired Wilcoxon Signed Rank test, excluding any missing data points for a given comparison. Outcome variables at the post-operative sixth month (Post-Op 6 months) were compared against baseline prior to prehabilitation (Week 0), two weeks after prehabilitation (Week 2), and post-operative first month (Post-Op 1 Month). Results that yield a *p*-value of <0.05 were regarded as statistically significant.

## 3. Results

### 3.1. Patient Characteristics

A total of 40 patients were recruited for the interventional cohort. After removing patients who were lost to follow-up, 36 patients were eligible for the HEROS study (Supplementary Figure S1). The compliance to post-operative HP ONS with HMB prescription was 80.5% (IQR = 33.3–96.9%). Baseline characteristics were reported in Table 1. The median age was 71.5 years, and 18 of the participants (50.0%) were male. In terms of physiological characteristics, the median body-mass index was 21.6 kg/m^2^, while 12 (33.3%) and 24 (66.7%) participants were assessed to have sarcopenia and severe sarcopenia, respectively, according to the AWGS 2019 diagnostic criteria. The median length-of-stay of the participants was 7 days (IQR: 6 to 10.75).

### 3.2. Long-Term Outcomes

The study outcomes for muscle quality, functional status, anthropometric measurements, nutritional status and quality of life are shown in Table 2. Outcomes measured at Post-Op 3 Months and Post-Op 6 Months, or three and six months after the operation, respectively, were compared with measurements taken at Week 0, or prior to the initiation of prehabilitation, Week 2, or after two weeks of prehabilitation, and Post-Op 1 Month.

### 3.3. Changes in Outcomes over Time

#### 3.3.1. Muscle Quality

**Figure 2:**
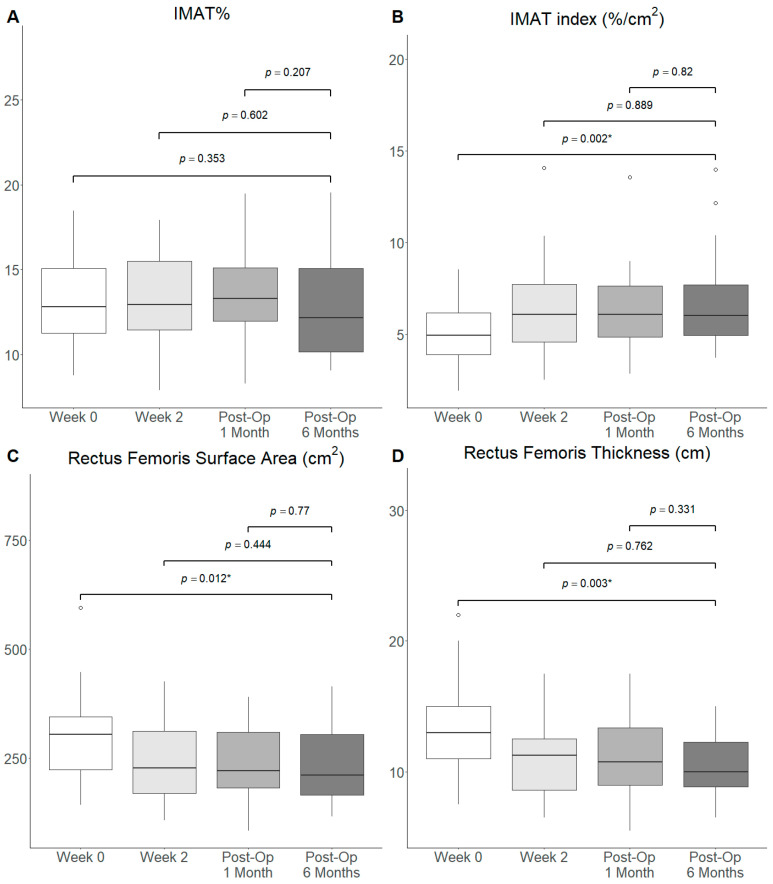
Box and whiskers plot of muscle quality measurements ((**A**) IMAT%, (**B**) IMAT index, (**C**) RFSA and (**D**) RFT) at baseline, Week 2, Post-Op 1 Month and Post-Op 6 Months. ◦: outlier >1.5 interquartile range from quartiles. *: *p*-value < 0.05.

There was a significant decrease in muscle quality as assessed by the IMAT index at Post-Op 6 Months when compared to Week 0 (Median: 6.0 vs. 4.9, *p* = 0.002), prior to initiation of the prehabilitation programme, but not with other timepoints (Figure 2). There was a significant decrease in RFSA (Median: 227 vs. 304, *p* = 0.012) and RFT (Median: 11 vs. 13, *p* = 0.003) observed at Post-Op 6 Months when compared to Week 0. IMAT% was not significantly different at Post-Op 6 Months compared to all other timepoints. Similarly, no significant difference in terms of the IMAT%, IMAT index, RFSA and RFT was observed when comparing Post-Op 3 Months with Week 0, Week 2 and Post-Op 1 Month (Supplementary Figure S2). IMAT index (Median: 6.0 vs. 4.9, *p* = 0.003), RFSA (Median: 205 vs. 295, *p* = 0.012) and RFT (Median: 10 vs. 13, *p* = 0.003) remained significantly different at Post-Op 6 months compared to Week 0 after outlier removal (Supplementary Figure S6).

#### 3.3.2. Functional Outcomes

**Figure 3:**
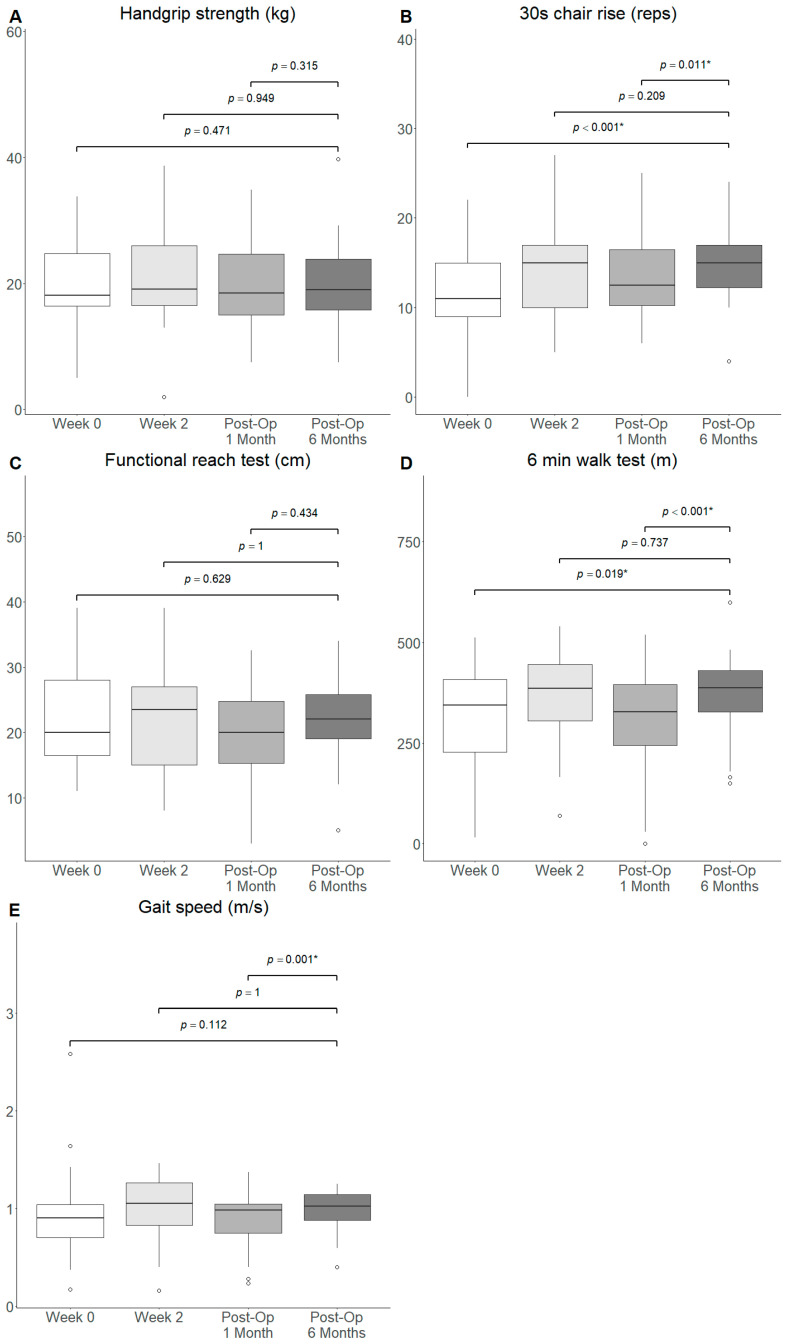
Box and whiskers plot of functional outcomes ((**A**) handgrip strength, (**B**) 30 s chair rise, (**C**) functional reach test, (**D**) 6MWT and (**E**) gait speed) from baseline, to Week 2, to Post-Op 6 Months. ◦: outlier >1.5 interquartile range from quartiles. *: *p*-value < 0.05.

No significant difference in handgrip strength, functional reach and gait speed was observed at Post-Op 6 Months from Week 0, Week 2 and Post-Op 1 Month of the prehabilitation programme, respectively (Figure 3A, Figure 3C, Figure 3E). The number of chair rise repetitions completed in 30 s was significantly increased at Post-Op 6 Months from Week 0 (Median: 15 vs. 11, *p* < 0.001) and Post-Op 1 Month (Median: 15.0 vs. 13.0, *p* = 0.011) but not from Week 2 (Figure 3B). Similarly, the 6MWT was significantly improved at Post-Op 6 Months compared to Week 0 (Median: 387 vs. 349, *p* = 0.020) and Post-Op 1 Month (Median: 387 vs. 336, *p* < 0.001) but not Week 2 (Figure 3D).

Similar findings were observed when comparing the functional outcomes at Post-Op 3 Months with Week 0 and Week 2 (Supplementary Figure S4). Significant improvements in the 30 s chair rise (Median: 14 vs. 11, *p* = 0.002; Median: 14 vs. 13, *p* = 0.002) and 6MWT (Median: 388 vs. 349, *p* = 0.026; Median: 388 vs. 336, *p* < 0.001) were observed at Post-Op 3 Months compared to Week 0 and Post-Op 1 Month. All other comparisons were insignificant.

Similar findings were observed in the sensitivity analysis, where outliers were removed when comparing functional outcomes at Post-Op 6 Months with Week 0, Week 2 and Post-Op 1 Month (Supplementary Figure S7). Chair rise repetitions within 30 s (Median: 15 vs. 11, *p* < 0.001) and 6MWT (Median: 399 vs. 354, *p* = 0.031) were significantly improved at Post-Op 6 months compared to baseline. Of note, chair rise repetitions (Median: 15 vs. 13, *p* = 0.006), 6MWT (Median: 399 vs. 368, *p* < 0.001), and gait speed (Median: 1.06 vs. 0.99, *p* = 0.003) significantly improved at Post-Op 6 Months when compared to Post-Op 1 Month. All other comparisons were insignificant.

#### 3.3.3. Anthropometric Measurements

**Figure 4:**
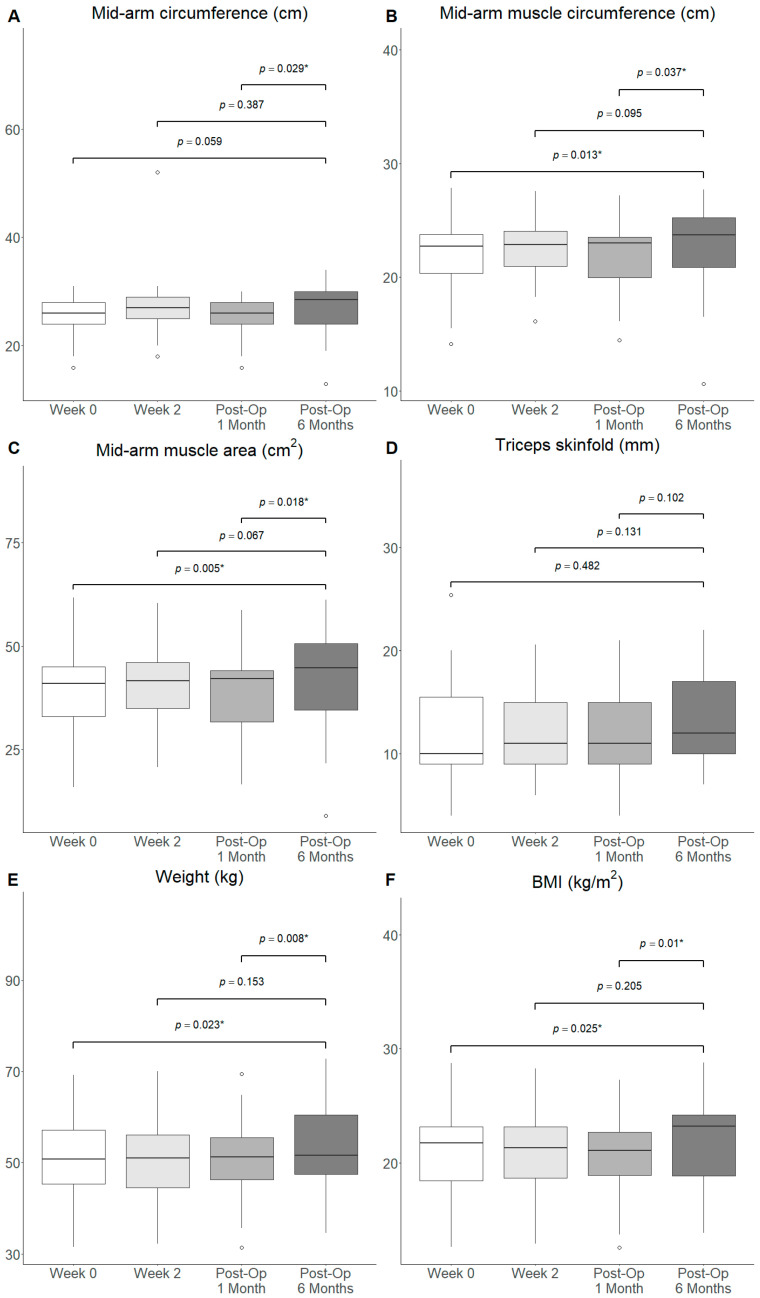
Box and whiskers plot of anthropometric measurements ((**A**) mid-arm circumference, (**B**) mid-arm muscle circumference, (**C**) mid-arm muscle area, (**D**) triceps skinfold, (**E**) weight and (**F**) BMI) at baseline, Week 2, Post-Op 1 Month and Post-Op 6 Months. ◦: outlier >1.5 interquartile range from quartiles. *: *p*-value < 0.05.

The mid-arm circumference (Median: 28.5 vs. 26.0, *p* = 0.029), mid-arm muscle circumference (Median: 23.7 vs. 23.2, *p* = 0.037) and mid-arm muscle area (Median: 44.8 vs. 42.8, *p* = 0.018) were increased significantly at Post-Op Month 6 from Post-Op Month 1 (Figure 4A–C). The mid-arm muscle circumference (Median: 23.7 cm vs. 22.5 cm, *p* = 0.013), mid-arm muscle area (Median: 44.8 cm^2^ vs. 39.3 cm^2^, *p* = 0.005), weight (Median: 51.6 kg vs. 50.8 kg, *p* = 0.023) and BMI (Median: 23.2 kg/m^2^ vs. 21.8 kg/m^2^, *p* = 0.025) increased significantly at Post-Op Month 6 from Week 0, but not from Week 2 (Figure 4B,C,E). Meanwhile, no significant differences were noted in the mid-arm circumference and triceps skinfold at Post-Op 6 Months when compared to Week 0 and Week 2 (Figure 4A,D). Meanwhile, no significant difference in all anthropometric measurements was observed at Post-Op 3 Months compared to Week 0, Week 2 and Post-Op 1 Month (Supplementary Figure S4).

In the sensitivity analysis where outliers were removed, mid-arm circumference (Median: 29.0 vs. 26.3, *p* = 0.034), mid-arm muscle circumference (Median: 23.8 vs. 22.9, *p* = 0.005), mid-arm muscle area (Median: 45.1 vs. 41.6, *p* = 0.003), weight (Median: 51.9 vs. 50.8, *p* = 0.038) and BMI (Median: 23.2 vs. 21.9, *p* = 0.038) were significantly greater at Post-Op 6 Months compared to baseline (Supplementary Figure S8). Mid-arm circumference (Median: 29.0 vs. 26.5, *p* = 0.012), mid-arm muscle circumference (Median: 23.8 vs. 23.2, *p* = 0.011), mid-arm muscle area (Median: 45.1 vs. 42.8, *p* = 0.006), weight (Median: 51.9 vs. 51.9, *p* = 0.020) and BMI (Median: 23.2 vs. 21.6, *p* = 0.016) were also significantly greater at Post-Op 6 Months compared to Post-Op 1 Month after outlier exclusion. All other comparisons were insignificant.

#### 3.3.4. Subjective Outcomes: Quality of Life and Nutritional Status

**Figure 5:**
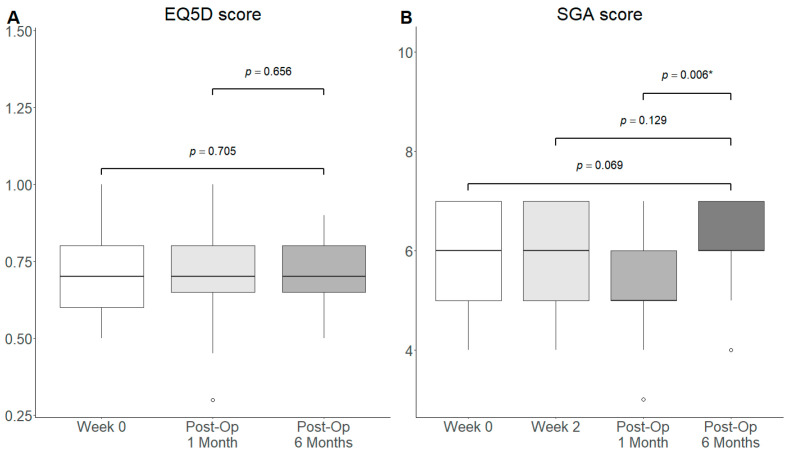
Box and whiskers plot of subjective outcomes ((**A**) quality of life using EQ5D and (**B**) nutritional status using SGA) at baseline, Week 2 (for nutritional status), Post-Op 1 Month and Post-Op 6 Months. ◦: outlier >1.5 interquartile range from quartiles. *: *p*-value < 0.05.

Nutritional status as measured by SGA score was significantly increased at Post-Op 6 Months compared to Post-Op 1 Month (Median: 6.0 vs. 5.0, *p* = 0.006). Otherwise, no significant difference in quality-of-life as measured using the EQ5D score and nutritional status using the SGA was observed at Post-Op 6 Months from Week 0 and Week 2 (Figure 5). This was also observed at Post-Op 3 Months from Week 0, Week 2 and Post-Op 1 Month (Supplementary Figure S5).

In the sensitivity analysis with outlier exclusion, nutritional status was significantly improved at Post-Op 6 Months (Median: 6.5 vs. 6.0, *p* = 0.044) compared to baseline and Post-Op 1 Month (Median: 6.5 vs. 5.5, *p* = 0.006). All other comparisons were insignificant. (Supplementary Figure S9).

## 4. Discussion

This study expands on the previous interim analysis and evaluates the impact of multimodal prehabilitation incorporating HP ONS with HMB and resistance exercise on sarcopenic surgical patients and its effect on muscle quality up to an extended duration of six months post-operation. We reported improvements in muscle quality as measured by the IMAT index, indices of functional status including gait speed, 30 s chair rise and 6MWT, anthropometric indices such as weight and mid-arm measurements, as well as nutritional status in the extended recovery period at six months post-operation compared to the acute window at one-month post-operation. In keeping with previously published findings [32], we reported improvements in the aforementioned outcomes following two weeks of prehabilitation, as comparisons were significant for measurements taken at six months post-operation and baseline. Specific to functional outcomes, observed improvements were at or exceeded previously reported minimal clinically important difference (MCID) thresholds. For example, patients post-operatively at 6 months were on average able to perform 2 more chair rises in 30 s, which is within the MCID range of 2.0 to 2.6 [50] and walk an additional distance of 51 m, exceeding the MCID range of 14 to 35 m [51]. This further suggests that improvements following prehabilitation were robust and persistent in the post-operative window that are potentially meaningful for the patient and outcomes.

Previous studies have demonstrated possible perioperative benefits in terms of reduced surgical complications and improved function amongst patients with prehabilitation [52,53,54]. However, there is limited evidence to date demonstrating the persistence of such benefits in the long-term past 30 days. The evidence for long-term benefits specific to resistance training and nutrient supplementation is further limited. Hence, our study reports, to our knowledge, the first demonstrated long-term benefit for short-term prehabilitation involving exercise and nutrition for sarcopenic patients following major surgeries.

Of note, functional and subjective outcomes improved significantly following two weeks of prehabilitation, indicating a relatively fast rate of gain. We hypothesize that this might be due to changes in muscle metabolism and biochemical processes in the perioperative phase that were optimised via nutrition and exercise. Notably, the supplement provided in this study, HMB, is an active metabolite in regulating muscle protein synthesis. It has been shown to inhibit ubiquitin-proteasome proteolytic pathways while also stimulating protein synthesis via the mTOR pathway [27]. In the older adult populations, age-induced mitochondrial dysfunction and subsequent reactive oxygen species-mediated damage are key pathophysiological pathways towards muscle atrophy and sarcopenia [55]. As such, the anti-apoptotic and proliferative effects of HMB can help buffer against age-related cellular changes that may otherwise lead to sarcopenia. Moreover, HMB has been shown to lower the levels of inflammatory cytokines like TNF-α and IL-6, which play a role in preventing muscle degradation [56,57]. Additionally, HMB may enhance mitochondrial function by increasing mitochondrial biogenesis, a process that is important for boosting energy production to maintain muscle function [58]. Coupled with resistance training, which has been demonstrated to prevent muscle atrophy and promote muscle growth in sarcopenic patients [55]. The synergistic effect of optimal nutrition with HMB and muscle mass maintenance and growth with exercise training might have contributed to the short-term gains in function and muscle quality. However, we further posit that due to the increased reserve built up in muscle quality and mass, as well as functional outcomes through the prehabilitation programme [59], the gradient and magnitude of decline in muscle quality and function were buffered against such that gains achieved during the prehabilitation programme were maintained in the postoperative period. This can be attributed to factors such as patient education during prehabilitation, leading to increased self-care [60,61], reduced surgical complications following preoperative optimisation contributed by prehabilitation [62,63], and direct attenuation of sarcopenic pathways in host muscle tissue [64,65]. However, we caution that given the observational nature of our study, our findings remain hypothesis-generating and the aforementioned mechanisms are grounds for further research.

Interestingly, two weeks of HMB supplementation during prehabilitation was associated with an increased IMAT index in older individuals [32]. There were three potential explanations for this observation. First, Impaired mitochondrial oxidation of Branched Chain Amino Acid (BCAA) in the muscle organ of older patients caused accumulation of HMB and leucine [66,67,68,69,70,71]. To date, leucine is recognised as one of the most important branched-chain amino acids (BCAAs), with HMB being a key metabolite derived from leucine. HMB could be directly converted to cholesterol and acetoacetyl-CoA for lipid synthesis rather than being effectively utilised through typical metabolic pathways, thereby promoting intramuscular adipose tissue (IMAT) accumulation [72,73]. Second, elevated HMB levels through supplementation may activate signalling pathways such as PI3K-AKT and mTOR, which favour adipogenesis, lipid synthesis, and subsequent lipid deposition within muscle tissue [74,75,76]. Third, despite the reduced rate of BCAA catabolism in skeletal muscle, particularly among older individuals, KIC (keto-isocaproate) derived from HMB may activate the branched-chain α-keto acid dehydrogenase (BCKD) complex, accelerating KIC catabolism and leading to the production of branched-chain fatty acids and acetyl-CoA for de novo lipogenesis [77,78]. This process ultimately promoted lipid accumulation within muscle tissue. Surgical stress increased energy demands by inducing acute metabolic stress, elevating stress hormones and inflammatory cytokines, thereby stimulating lipolysis and mobilising intramuscular fat reserves to meet immediate energy requirements. Consequently, the IMAT index decreased during the surgical phase. In older adults, despite initial reductions during acute stress, IMAT index re-accumulates by Post-Op 6 months following HMB supplementation. This rebound phenomenon results from persistent anabolic resistance, altered metabolic fate of HMB metabolites toward lipid synthesis, decreased physical activity, and hormonal/inflammatory shifts conducive to intramuscular adipogenesis [79,80]. We hypothesise that energy in older individuals with poorer muscular regenerative ability is stored in fat rather than seen as an improvement of muscle quality, especially in the short term. This is thereby reflected in an increase in the IMAT index. As the muscle fat in individuals increased, the functional and anthropometric measurements also appeared to similarly improve. Hence, our study suggests possible age-specific changes in muscle quality, although this has to be interpreted with caution, given the lack of prevailing consensus in the understanding of muscle quality.

It was noted that the rectus femoris surface area and thickness significantly decreased, which may suggest that the changes in IMAT index observed herein can be partially attributed to smaller muscle size. It is well-recognised that muscle size and mass generally decrease postoperatively [81,82,83] with advanced age, male gender and preoperative comorbidities as significant risk factors [84]. Varying extents of postoperative muscle loss are unavoidable due to protein catabolism following surgical stress [85,86], poor nutrition from limited oral intake [87,88] and disuse atrophy due to immobilisation [89]. Muscle quality was also observed to decrease postoperatively in other studies [90,91], which in turn is associated with poorer function and more complicated recovery [92,93]. Molinger et al. (2024) noted that a slight decrease in muscle area and muscle thickness, combined with increased intramuscular lipid accumulation, could lead to a higher IMAT index in ICU patients from admission to discharge [94]. The combination of these factors could account for the reduced IMAT index in our patients. However, in our study, while the rectus femoris surface area and thickness decreased, the mid-arm circumference and mid-arm surface area increased instead. Differential usage between upper and lower limbs post-operatively may account for these differences. Additionally, despite the reported decrease in muscle quality, there was no apparent functional loss noted, if any. In fact, certain functional outcomes appear to improve from baseline. This suggests that muscle loss and deterioration post-operatively may not be global. The lack of functional decline indicates that prehabilitation intervention likely improved the recovery outcomes amongst these sarcopenic surgical patients in spite of muscle loss. Potential reasons for preservation of function in spite of reduced muscle volume and surface area include selective loss of adipose tissue without muscle breakdown [95] and learning of compensatory manoeuvres for more efficient muscle locomotion and function during physiotherapy [96,97].

The effects of the prehabilitation-rehabilitation continuum were seen to be long-lasting and sustained to 6 months post-operatively, despite the rehabilitation phase being stopped 2 months post-surgery. The magnitude of increase in BMI and muscle volume in terms of mid-arm muscle circumference and mid-arm muscle area was greater between Post-Op 6 Months and Week 0 than between Week 2 and Week 0. This suggested that not only was the gain in muscle volume and weight achieved from prehabilitation preserved over the six months, but the gain was compounded over time. In a recent study, the acute trajectory of recovery to baseline was significantly faster for the prehabilitation group [98]. Our study extends this observation, suggesting that this rate of recovery was potentially translated into sustained functional gains. Physiological recovery might be a plausible factor, as seen in healthy patients who experience acute muscle and strength loss following significant surgical stress that then return to baseline or even above after a few months [99,100]. However, such physiological recovery trajectories in muscle mass, strength and function may be absent or even follow a pattern of deterioration amongst sarcopenic and or older adult patients [101,102], as reported in previous studies. Yet, our study reported variations in muscle quality, muscle mass and functional outcomes amongst such sarcopenic older adult patients following HP ONS with HMB, which suggests a possible modality to track prehabilitative and post-operative status of muscle health. HMB may possibly provide the protective effect of exercise and nutrition-focused prehabilitation against sarcopenic decline following significant surgical stress. Similarly, the IMAT index was maintained through the timepoints, implying that the storage of energy in fat was also sustained, which may potentially be responsible for these sustained changes.

The demonstration of long-term benefits following prehabilitation prior to surgery in muscle mass and functional outcomes has clinical significance and real-world implications. Firstly, muscle mass has been established as a strong prognosticator of mortality and morbidity in various major surgeries [103,104,105,106,107]. In fact, a systematic review of 24 studies found that sarcopenia was associated with 1.61, 1.45 and 1.25 times lower chances of 1-year, 3-year and 5-year mortality [104]. Interestingly, a recent study showcased that pre-operative muscle mass influences long-term survival but not post-operative morbidity [108]. Secondly, perioperative sarcopenic states were found to be associated with poorer recovery of physical function [101], which then significantly limits the quality-of-life and independence of the patient postoperatively [109,110]. This also translates to poorer psychosocial functioning and distress in caregivers of such postoperative patients [111,112], which then leads to caregiver burnout [113], poorer coping in the caregiver-patient dyad [114] and adverse outcomes in the patient [115]. Hence, by implementing prehabilitation, adverse recovery trajectories can be avoided in the sarcopenic older adults, reducing morbidity and mortality while enhancing quality-of-life, independence and caregiver functioning. This then translates to cost savings as subsequent healthcare services utilisation and community burden are reduced.

Although our study demonstrated the effectiveness of exercise and nutrition-centred prehabilitation in achieving long-term muscle and functional gains, there are some limitations. Firstly, the present study is limited by a low sample size as it is a secondary analysis of long-term outcomes amongst a small cohort recruited for a proof-of-concept one-arm pilot interventional trial. Together with the ethnic homogeneity and restriction to elective gastrointestinal surgical patients, this results in a limited generalisability to other surgical populations, emergency surgery and non-Asian cohorts. As such, our findings might need further corroboration through larger-scale trials involving more participants from diverse backgrounds, as well as comparison with a control group without prehabilitation, to more definitively assign a causal effect of multimodal prehabilitation on muscle quality and functional outcomes. Furthermore, given the complex nature of the intervention with multiple modalities, it may be difficult to attribute the changes in muscle quality, function and quality-of-life observed in this study to HMB supplementation alone. Secondly, given that this is an intervention study with observed improvements in another muscle mass proxy (mid-arm circumference and mid-arm muscle area), relying solely on the rectus femoris muscle may not fully represent the overall muscle quality of individual patients. Lastly, the study is limited by the lack of data on perioperative characteristics of the recruited surgical patients, such as the specific type of surgery, site of surgery, intraoperative complications and so on. Such factors might influence the postoperative recovery trajectories in muscle and function, which we were not able to evaluate. Otherwise, the study attempted to control for confounders by adopting strict inclusion and exclusion criteria to ensure only elective gastrointestinal surgical patients were evaluated and had fair physical function preoperatively. Furthermore, the median compliance to HP ONS with HMB was similar to an average of 80.2% across 5 studies implementing multimodal prehabilitation in a systematic review [116]. While we did not investigate the influence of compliance, a high compliance rate for the post-operative HP ONS could potentially have contributed to the pronounced functional gains we noted, further emphasising the importance of the intervention’s duration and adherence to the regime.

### Future Perspectives

Future research should evaluate long-term outcomes through more frequent longitudinal assessments in larger cohorts, ideally utilising control groups without prehabilitation or isolated post-operative rehabilitation. Such designs would better characterise the trajectory of modifications to musculoskeletal status and physical performance. Additionally, isolating the prehabilitation components—such as nutrition-only versus exercise-only protocols—can also further elucidate the potential synergistic effects of multimodal intervention. Finally, incorporating advanced imaging modalities, such as a whole-body MRI, may provide a more comprehensive quantification of muscle quality and myosteatosis beyond the current pilot findings.

## 5. Conclusions

Multimodal prehabilitation involving HP ONS with HMB and resistance exercise for sarcopenic older adult patients undergoing elective gastrointestinal surgery was associated with gains in physical function preserved up to 6 months. However, given no significant changes to IMAT% were observed, these findings suggest that functional gains may occur independently of detectable changes in muscle quality in this small cohort. There is a potential that multimodal prehabilitation programmes centred on exercise and nutrition can be used to optimise postoperative recovery trajectories towards regaining function amongst sarcopenic older adults undergoing surgery. However, further large-scale controlled trials are necessary to better characterise these changes and determine the long-term impact on post-operative outcomes.

## Figures and Tables

**Table 1:** Baseline patient characteristics of 36 recruited participants.

Patient Characteristics	Total, N = 36
**Demographics**	
Age, in years (range)	71.5 (55–90)
Male, n (%)	18 (50%)
**Race**	
Chinese, n (%)	30 (83.3%)
Malay, n (%)	4 (11.1%)
Indian, n (%)	2 (5.6%)
**Anthropometrics**	
Body-mass-index (kg/m^2^)	21.6 (12.9–28.4)
Height (m)	1.56 (1.40–1.69)
Weight (kg)	51.0 (32.2–70.2)
**Sarcopenia status**	
Sarcopenia, n (%)	12 (33.3%)
Severe sarcopenia, n (%)	24 (66.7%)
**Sarcopenia diagnostic measures**	
Handgrip strength (kg)	18.1 (5.0–33.8)
6MWT (m/s)	0.9 (0.17–2.58)
Bioelectrical impedance analysis (kg/m^2^)	5.76 (3.93–6.9)

**Table 2:** Study outcomes at timepoints Week 0, Week 2, Post-Op 1 Month, Post-Op 3 Months and Post-Op 6 Months. All variables are presented in median (IQR).

Outcome	Timepoint				
	Week 0	Week 2	Post-Op 1 Month	Post-Op 3 Months	Post-Op 6 Months
**Muscle quality**					
IMAT%	12.8 (11.3–15.1)	13.0 (11.5–15.5)	13.5 (12.0–15.1)	12.7 (10.6–15.2)	12.2 (10.2–15.1)
IMAT index (%/cm^2^)	4.9 (3.9–6.1)	6.1 (4.6–7.7)	6.1 (4.8–7.5)	5.3 (4.4–6.5)	6.0 (4.9–7.7)
Rectus femoris surface area (cm^2^)	304 (223–344)	221 (182–310)	233 (199–314)	211 (166–304)	227 (169–313)
Rectus femoris thickness (cm)	13 (11–15)	11 (9–14)	12 (10–13)	10 (8.9–12)	11 (8.6–13)
**Functional status**					
Handgrip strength (kg)	18.1 (16.5–24.3)	19.1 (16.5–26.0)	18.6 (16.8–25.0)	20.0 (15.9–23.9)	19.0 (15.8–23.9)
30 s chair rise (reps)	11.0 (9–15.5)	15.0 (10.0–17.0)	13.0 (11.8–17.0)	14.0 (12.5–16)	15.0 (12.3–17.0)
Functional reach test (cm)	20.5 (16.6–28.8)	23.5 (15.0–27.0)	20.5 (15.8–25.3)	22.0 (19.0–25.0)	22.0 (19.0–25.8)
6MWT (m)	349 (233–431)	386.0 (306–446)	336 (272–399)	388.0 (308–439)	387 (328–431)
Gait speed (m/s)	0.9 (0.7–1.0)	1.1 (0.8–1.3)	1.0 (0.9–1.1)	1.1 (0.8–1.3)	1.0 (0.9–1.1)
**Anthropometric measurements**					
Mid-arm circumference (cm)	26.0 (24.0–28.0)	27.0 (25.0–29.0)	26.0 (24.0–28.0)	26.0 (24.0–29.0)	28.5 (24.0–30.0)
Mid-arm muscle circumference (cm)	22.5 (20.2–23.6)	22.9 (21.0–24.0)	23.2 (20.1–23.6)	22.7 (20.6–23.7)	23.7 (20.9–25.2)
Mid-arm muscle area (cm^2^)	39.3 (32.6–44.3)	41.6 (35.0–46.0)	42.8 (32.3–44.2)	40.9 (33.6–44.6)	44.8 (34.6–50.7)
Triceps skinfold (mm)	10.0 (9.0–15.3)	11.0 (9.0–15.0)	11.0 (9.0–15.0)	11.0 (10.0–17.0)	12.0 (10.0–17.0)
Weight (kg)	50.8 (45.2–57.0)	51 (44.5–56.1)	51.9 (47.0–56.0)	51.9 (48.9–57.1)	51.6 (47.5–60.5)
Body Mass Index (kg/m^2^)	21.8 (18.6–23.1)	21.3 (18.7–23.2)	21.1 (18.9–22.7)	21.9 (18.6–24.1)	23.2 (18.9–24.2)
**Subjective Outcomes**					
Nutritional Status (SGA score)	6.0 (5.0–7.0)	6.0 (5.0–7.0)	5.0 (5.0–6.0)	6.0 (5.0–7.0)	6.0 (6.0–7.0)
Quality of life (EQ5D-3L)	0.70 (0.60–0.80)	NA	0.70 (0.65–0.80)	0.70 (0.60–0.75)	0.70 (0.65–0.79)

NA: there was no collection for EQ5D at that timepoint.

## Data Availability

Data described in the manuscript, code book, and analytic code will be made available upon reasonable request to the corresponding author due to restrictions (due to prevailing local PDPA laws by the government of Singapore, all request for data must go through the principal investigator and specific data in an anonymised fashion can then be provided with a signed agreement from the requestor).

## Supplementary Materials

The following supporting information can be downloaded at: https://www.mdpi.com/article/10.3390/nu18040703/s1, Supplementary Figure S1: Flowchart of patient inclusion and exclusion; Supplementary Figure S2: Box and whiskers plot of muscle quality measurements (1A. IMAT%, 1B. IMAT index, 1C. RFSA and 1D. RFT) at baseline, Week 2, Post-Op 1 Month and Post-Op 3 Months. ◦: outlier >1.5 interquartile range from quartiles. *: *p*-value < 0.05; Supplementary Figure S3: Box and whiskers plot of functional outcomes (2A. handgrip strength, 2B. 30 s chair rise, 2C. functional reach test, 2D. 6MWT and 2E. gait speed) at baseline, Week 2, Post-Op 1 Month and Post-Op 3 Months ◦: outlier >1.5 interquartile range from quartiles. *: *p*-value < 0.05; Supplementary Figure S4: Box and whiskers plot of anthropometric measurements (3A. mid-arm circumference, 3B. mid-arm muscle circumference, 3C. mid-arm muscle area, 3D. triceps skinfold, 3E. weight and 3F. BMI) atbaseline, Week 2, Post-Op 1 Month and Post-Op 3 Months. ◦: outlier >1.5 interquartile range from quartiles. *: *p*-value < 0.05; Supplementary Figure S5: Box and whiskers plot of subjective outcomes (4A. quality of life using EQ5D and 4B. nutritional status using SGA) at baseline, Week 2 (for nutritional status), Post-Op 1 Month and Post-Op 3 Months. ◦: outlier >1.5 interquartile range from quartiles. *: *p*-value < 0.05; Supplementary Figure S6: Box and whiskers plot of muscle quality measurements (5A. IMAT%, 5B. IMAT index, 5C. RFSA and 5d. RFT) at baseline, Week 2, Post-Op 1 Month and Post-Op 6 Months, with outliers two standard deviations above and below the mean removed. ◦: outlier >1.5 interquartile range from quartiles. *: *p*-value < 0.05; Supplementary Figure S7: Box and whiskers plot of functional outcomes (6A. handgrip strength, 6B. 30 s chair rise, 6C. functional reach test, 6D. 6MWT and 6E. gait speed) at baseline, Week 2, Post-Op 1 Month and Post-Op 6 Months with outliers two standard deviations above and below the mean removed ◦: outlier >1.5 interquartile range from quartiles. *: *p*-value < 0.05; Supplementary Figure S8: Box and whiskers plot of anthropometric measurements (7A. mid-arm circumference, 7B. mid-arm muscle circumference, 7C. mid-arm muscle area, 7D. triceps skinfold, 7E. weight and 7F. BMI) atbaseline, Week 2, Post-Op 1 Month and Post-Op 6 Months with outliers two standard deviations above and below the mean removed. ◦: outlier >1.5 interquartile range from quartiles. *: *p*-value < 0.05; Supplementary Figure S9: Box and whiskers plot of subjective outcomes (5A. quality of life using EQ5D and 5B. nutritional status using SGA) at baseline, Week 2 (for nutritional status), Post-Op 1 Month and Post-Op 6 Months with outliers two standard deviations above and below the mean removed. ◦: outlier >1.5 interquartile range from quartiles. *: *p*-value < 0.05.

## Abbreviations

The following abbreviations are used in this manuscript:

AWGC	Asian Workgroup of Cachexia
AWGS	Asian Workgroup of Sarcopenia
6MWT	6-min walk test
EQ5D-3L	EuroQol-5 Dimension-3 Level
HMB	β-hydroxy β-methylbutyric acid
HP-ONS	high protein oral nutritional supplementation
IMAT	intramuscular adipose tissue
RFSA	rectus femoris surface area
RFT	rectus femoris thickness
SGA	Subjective Global Assessment
