# Supplementary material for: Long-Term Outcomes of Multimodal Prehabilitation with High Protein Oral and HMB Supplementation in Sarcopenic Surgical Patients: The HEROS Study
Source: Nutrients. 2026 Feb 22;18(4):703. doi: 10.3390/nu18040703 (PMC12943104; doi:10.3390/nu18040703)

Supplementary Figures and Tables

Supplementary Figure S1: Flowchart of patient inclusion and exclusion.

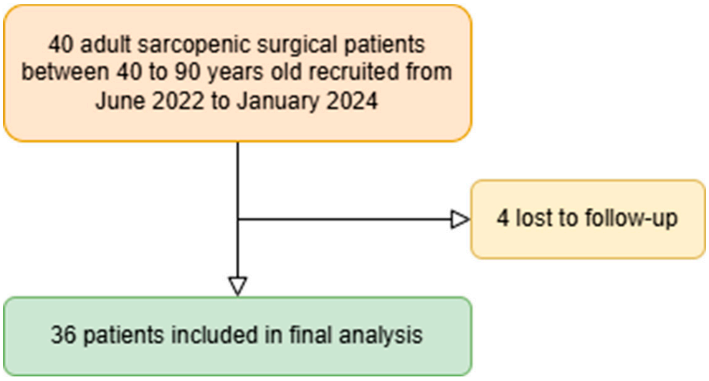

Supplementary Figure S2: Box and whiskers plot of muscle quality measurements (1a. IMAT%, 1b. IMAT index, 1c. RFSA and 1d. RFT) at baseline, Week 2, Post-Op 1 Month and Post-Op 3 Months. ◦: outlier >1.5 interquartile range from quartiles.*: *p*-value <0.05.

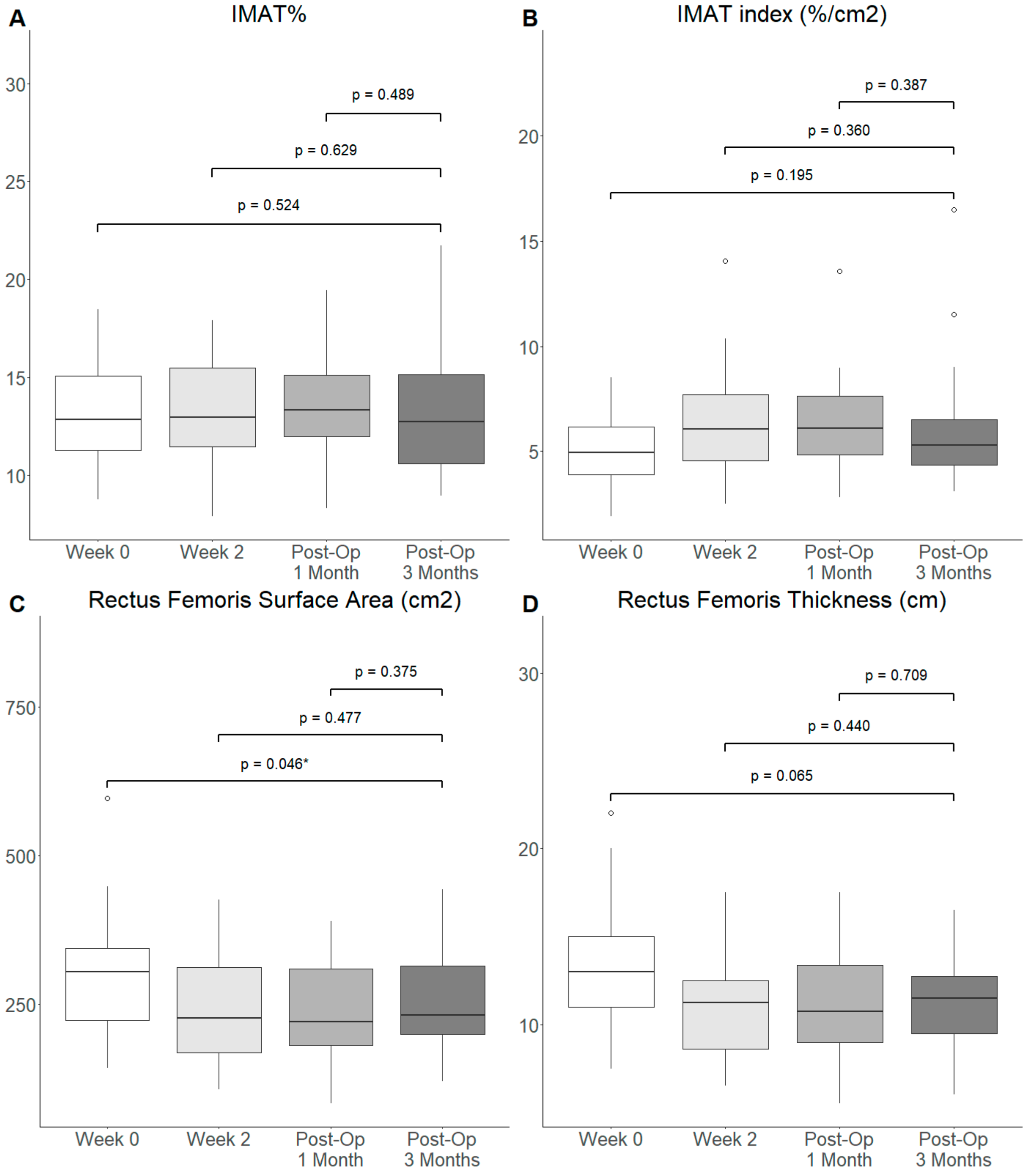

Supplementary Figure S3: Box and whiskers plot of functional outcomes (2a. handgrip strength, 2b. 30s chair rise, 2c. functional reach test, 2d. 6 min walk test and 2e. gait speed) at baseline, Week 2, Post-Op 1 Month and Post-Op 3 Months ◦: outlier >1.5 interquartile range from quartiles.*: *p*-value <0.05.

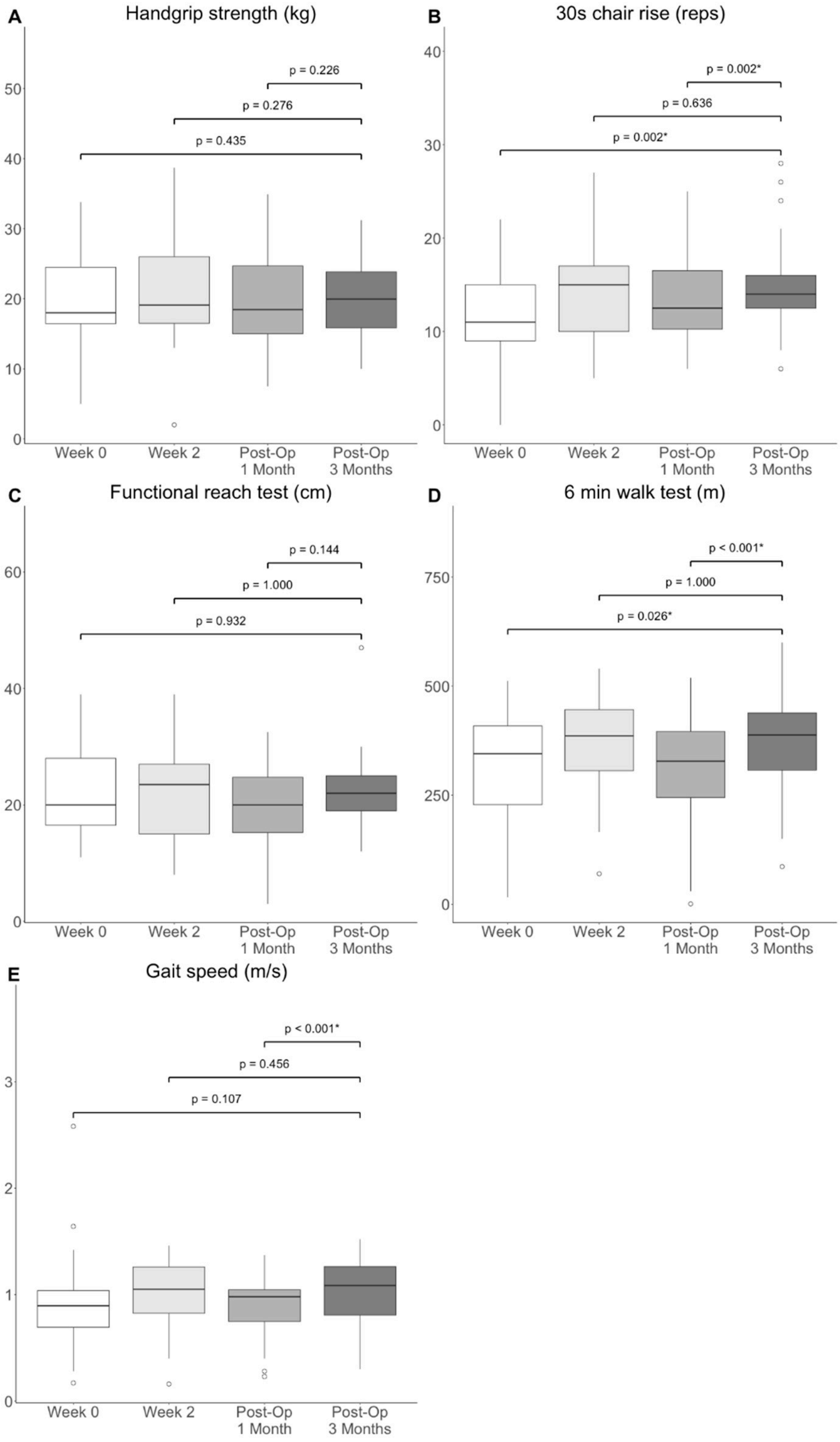

Supplementary Figure S4: Box and whiskers plot of anthropometric measurements (3a. mid-arm circumference, 3b. mid-arm muscle circumference, 3c. mid-arm muscle area, 3d. triceps skinfold, 3e. weight and 3f. BMI) atbaseline, Week 2, Post-Op 1 Month and Post-Op 3 Months. ◦: outlier >1.5 interquartile range from quartiles.*: *p*-value <0.05.

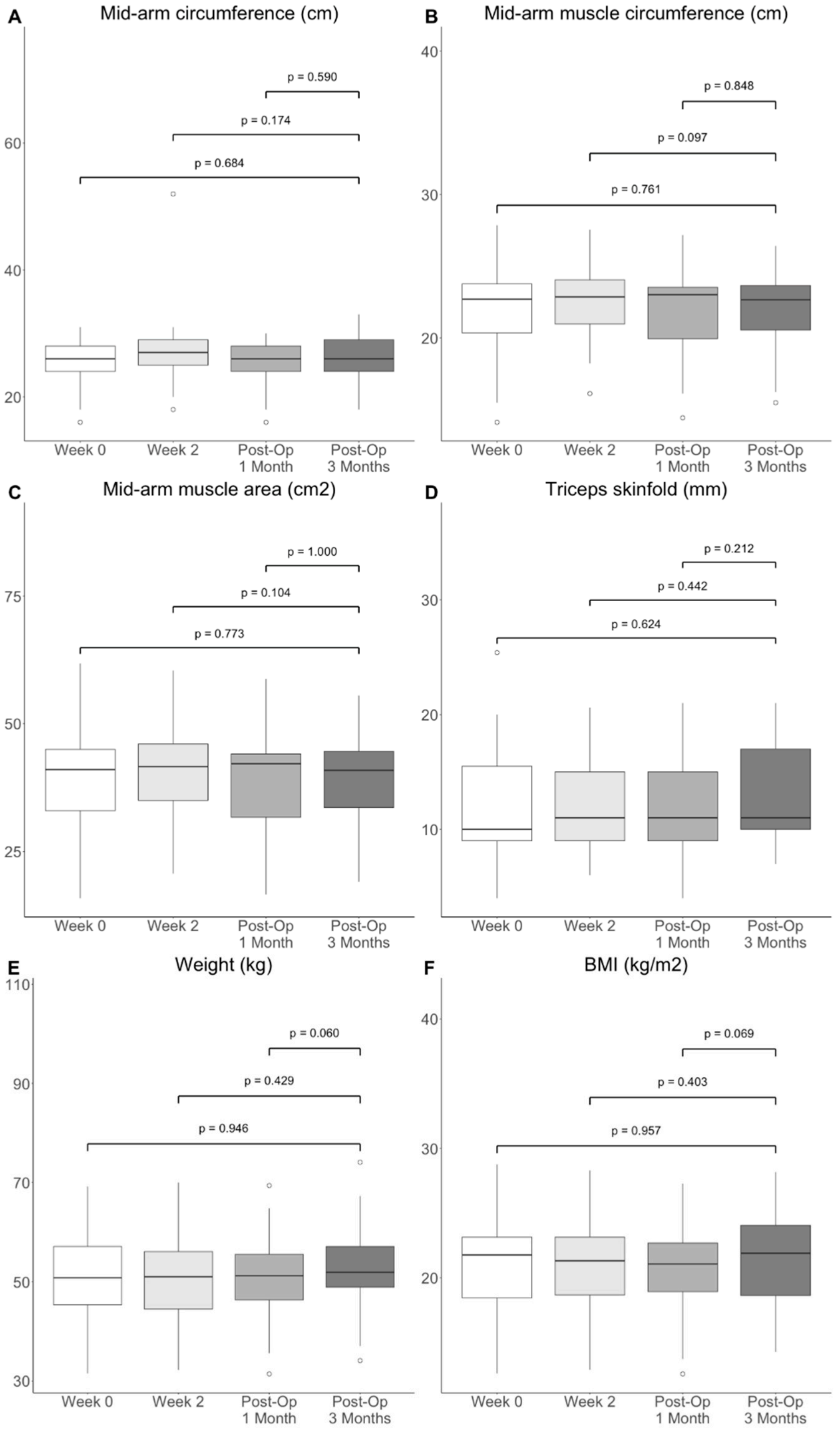

Supplementary Figure S5: Box and whiskers plot of subjective outcomes (4a. quality of life using EQ5D and 4b. nutritional status using SGA) at baseline, Week 2 (for nutritional status), Post-Op 1 Month and Post-Op 3 Months. ◦: outlier >1.5 interquartile range from quartiles. *: *p*-value <0.05.

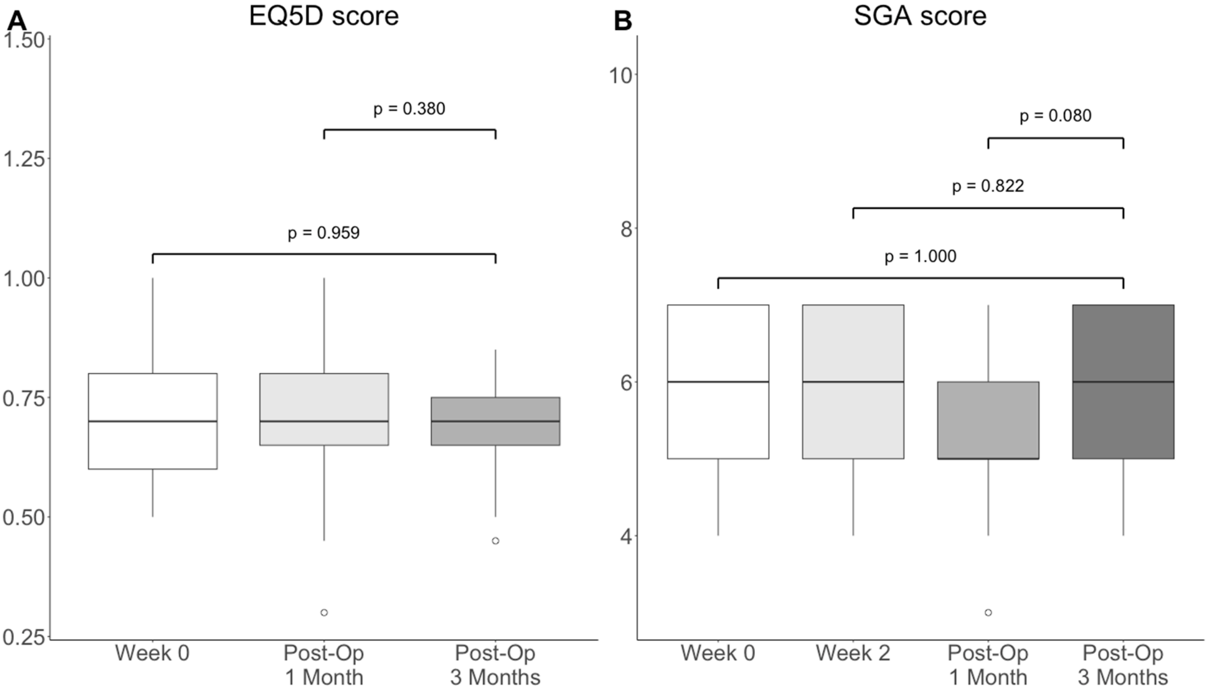

Supplementary Figure S6: Box and whiskers plot of muscle quality measurements (5a. IMAT%, 5b. IMAT index, 5c. RFSA and 5d. RFT) at baseline, Week 2, Post-Op 1 Month and Post-Op 6 Months with outliers two standard deviations above and below the mean removed. ∘: outlier >1.5 interquartile range from quartiles.*: *p*-value <0.05.

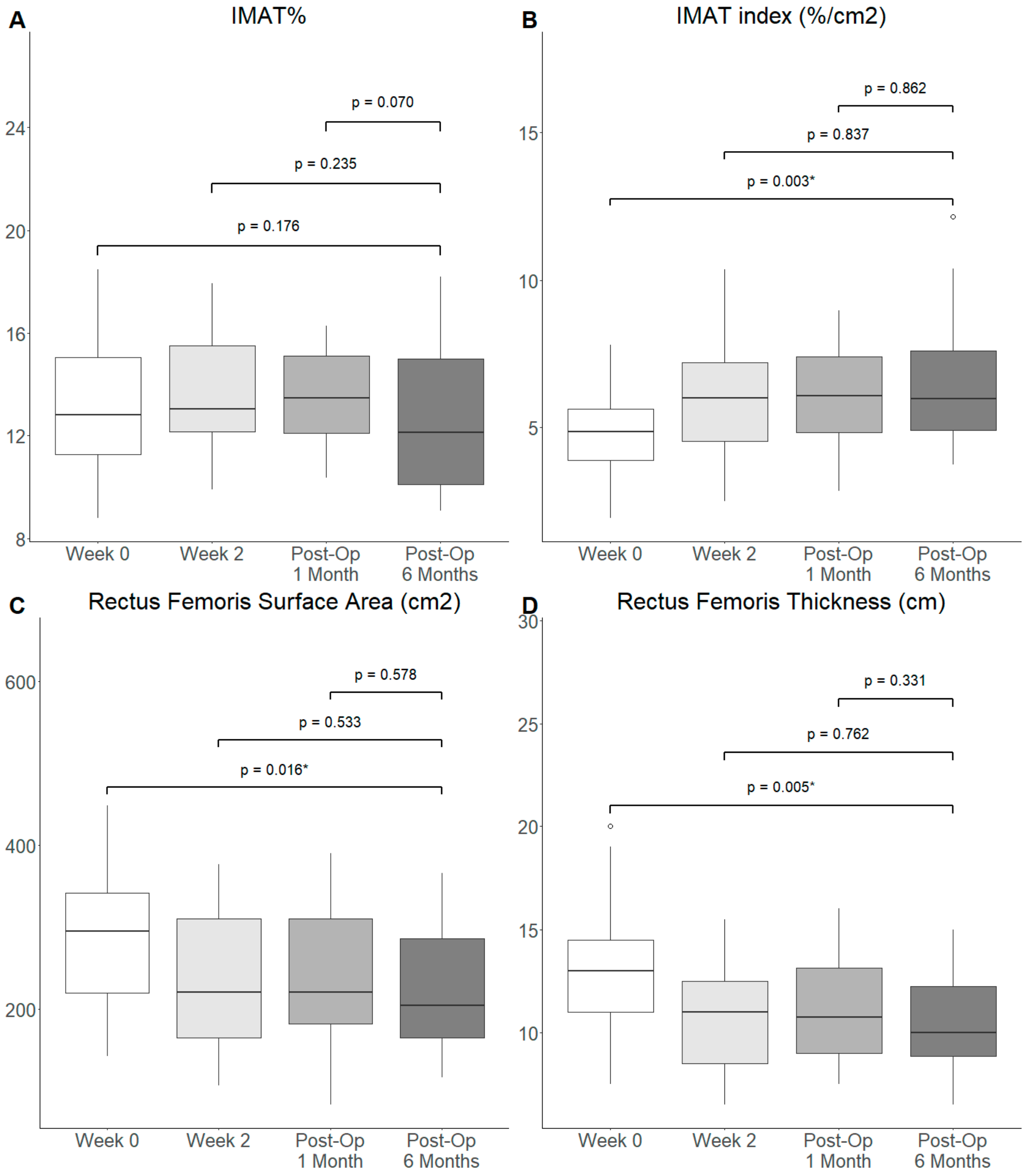

Supplementary Figure S7: Box and whiskers plot of functional outcomes (6a. handgrip strength, 6b. 30s chair rise, 6c. functional reach test, 6d. 6 min walk test and 6e. gait speed) at baseline, Week 2, Post-Op 1 Month and Post-Op 6 Months with outliers two standard deviations above and below the mean removed ◦: outlier >1.5 interquartile range from quartiles.*: p-value <0.05.

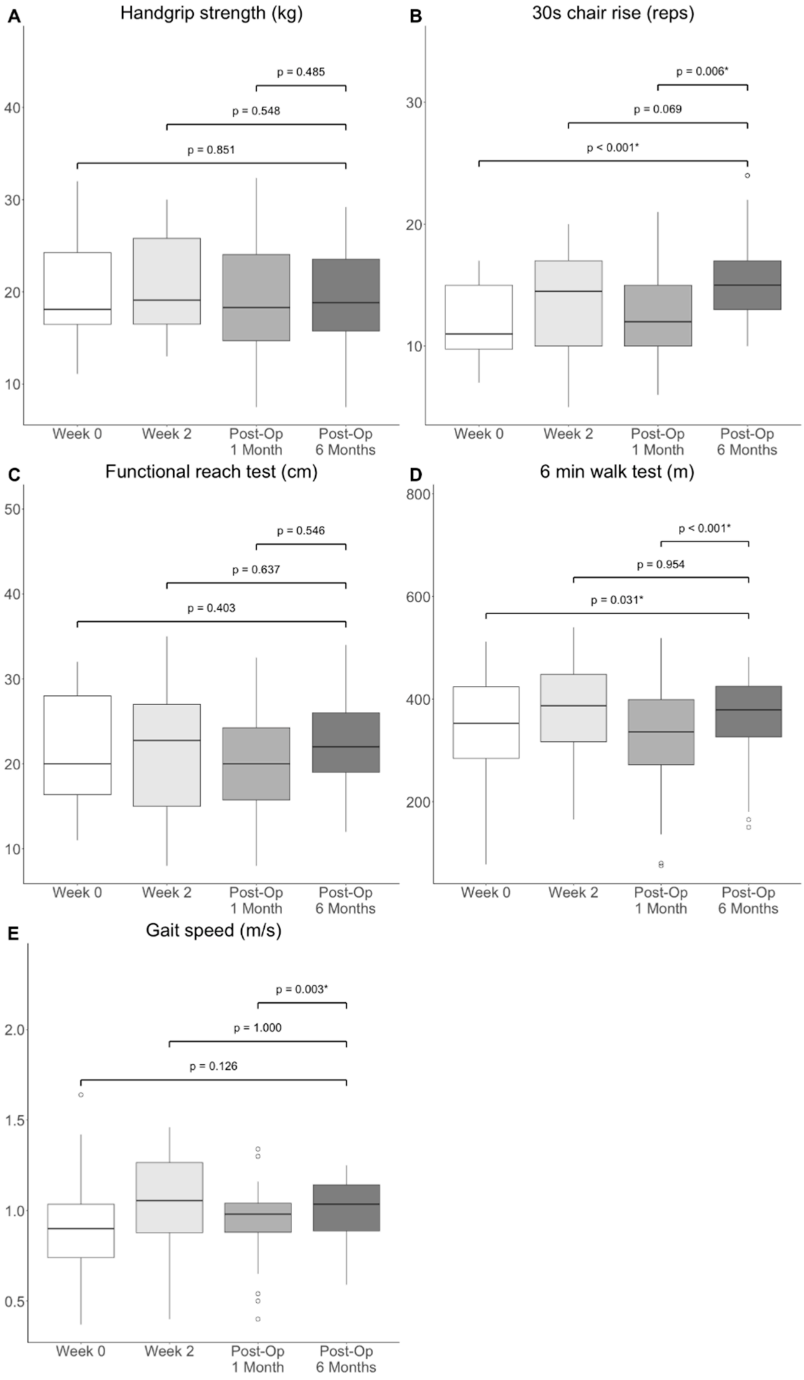

Supplementary Figure S8: Box and whiskers plot of anthropometric measurements (7a. mid-arm circumference, 7b. mid-arm muscle circumference, 7c. mid-arm muscle area, 7d. triceps skinfold, 7e. weight and 7f. BMI) atbaseline, Week 2, Post-Op 1 Month and Post-Op 6 Months with outliers two standard deviations above and below the mean removed. ◦: outlier >1.5 interquartile range from quartiles.*: *p*-value <0.05.

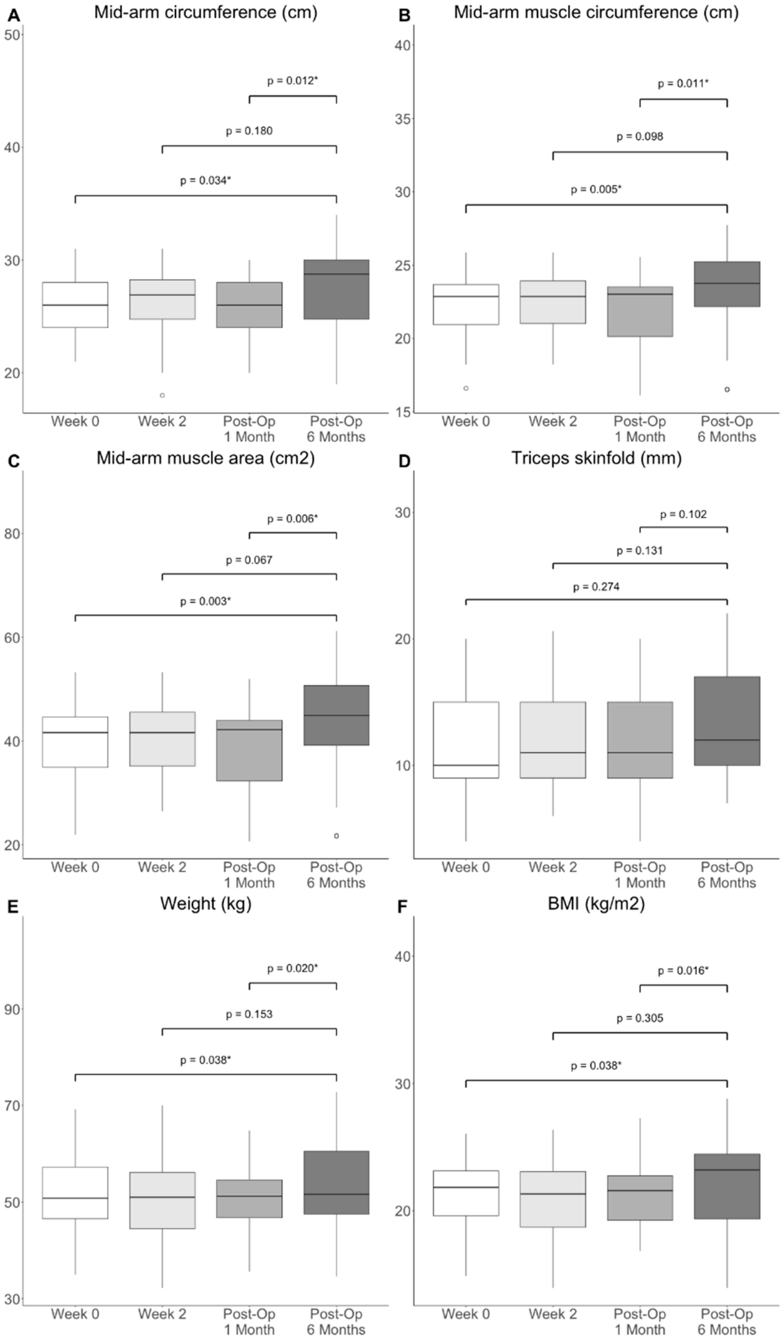

Supplementary Figure S9: Box and whiskers plot of subjective outcomes (5a. quality of life using EQ5D and 5b. nutritional status using SGA) at baseline, Week 2 (for nutritional status), Post-Op 1 Month and Post-Op 6 Months with outliers two standard deviations above and below the mean removed. ◦: outlier >1.5 interquartile range from quartiles. *: p-value <0.05.

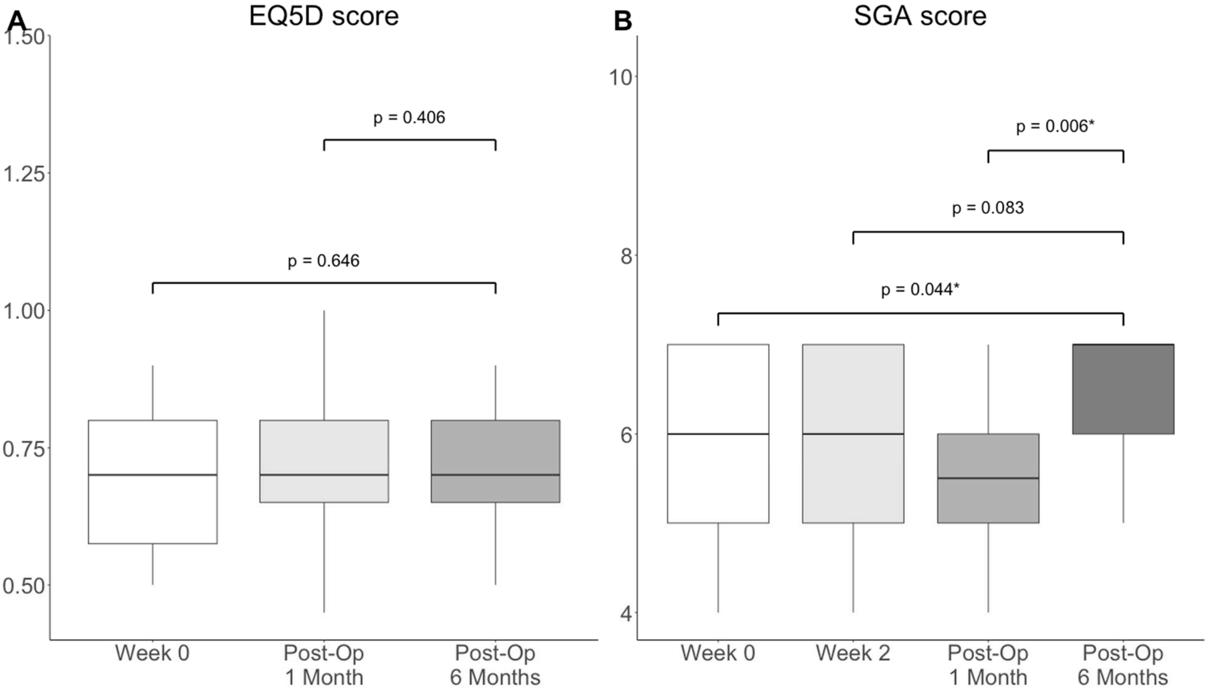

Supplement: Supplementary file 1 [file nutrients-18-00703-s001.zip › ESM.pdf]
