# Supplementary figures and images for: Long-Term Outcomes of Multimodal Prehabilitation with High Protein Oral and HMB Supplementation in Sarcopenic Surgical Patients: The HEROS Study
Source: Nutrients. 2026 Feb 22;18(4):703. doi: 10.3390/nu18040703 (PMC12943104; doi:10.3390/nu18040703)

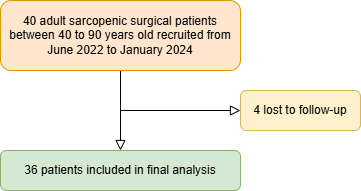

Supplement: Supplementary file 1 [file nutrients-18-00703-s001.zip › Supplementary Figure S1.png]

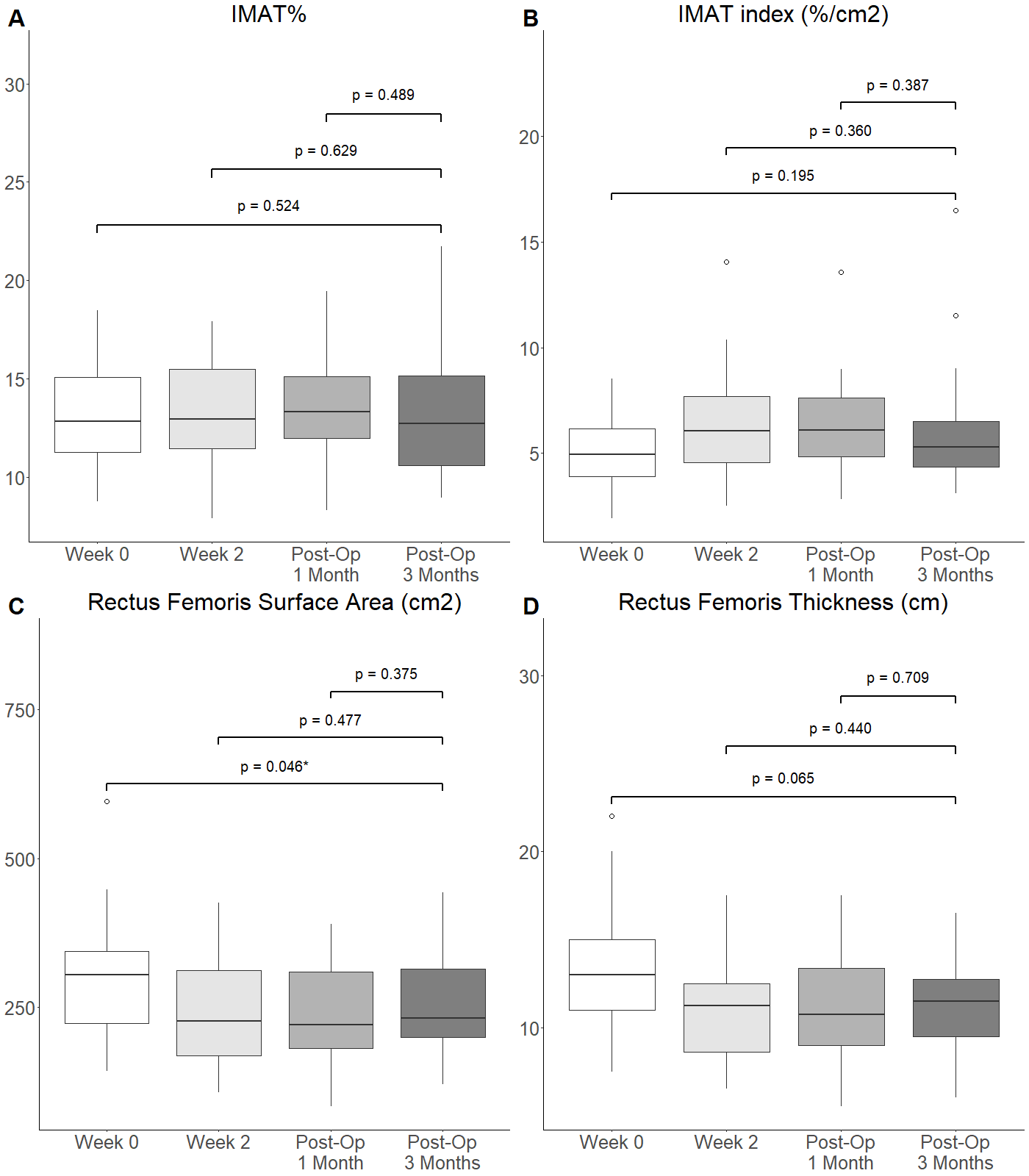

Supplement: Supplementary file 1 [file nutrients-18-00703-s001.zip › Supplementary Figure S2.png]

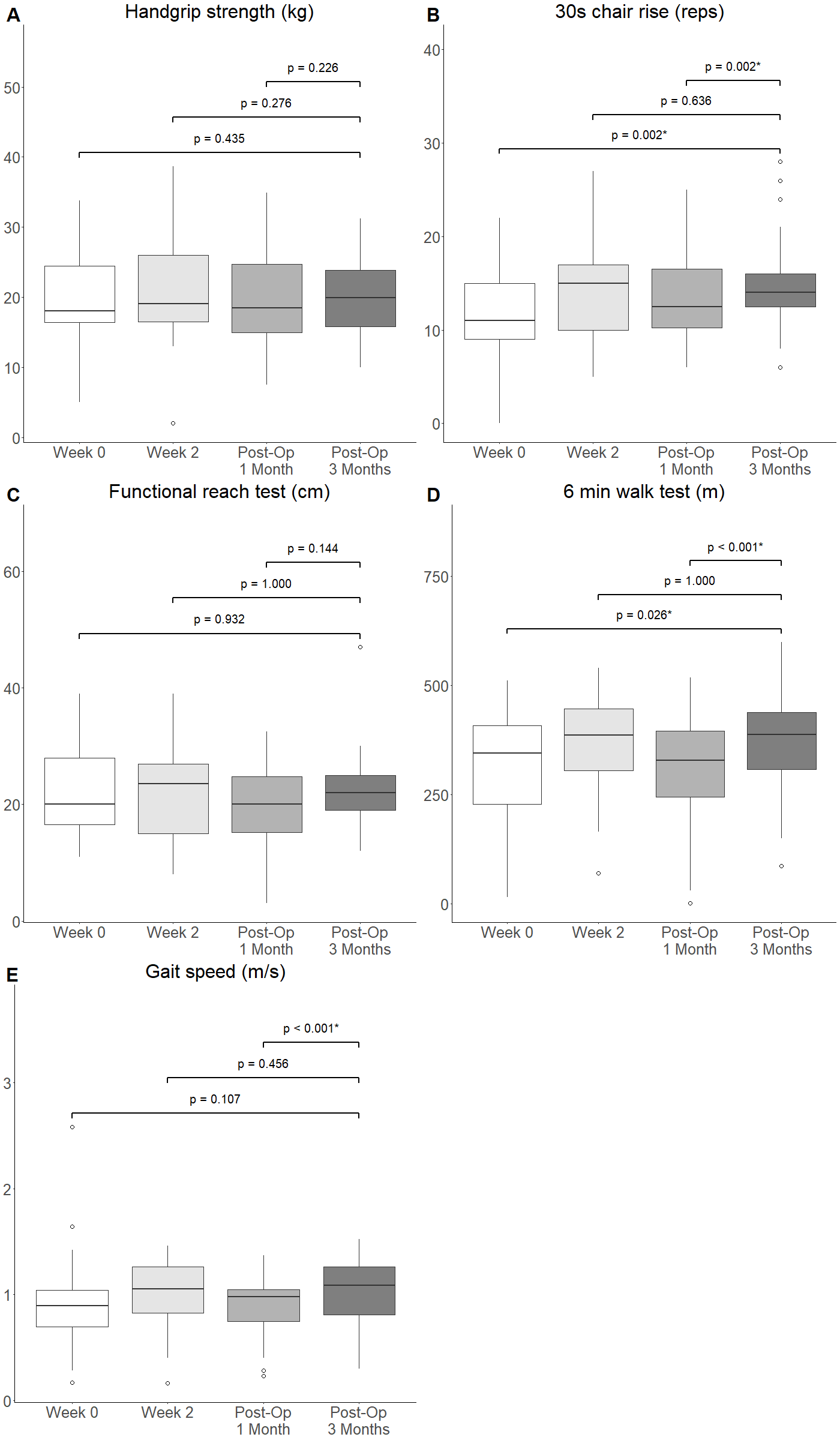

Supplement: Supplementary file 1 [file nutrients-18-00703-s001.zip › Supplementary Figure S3.png]

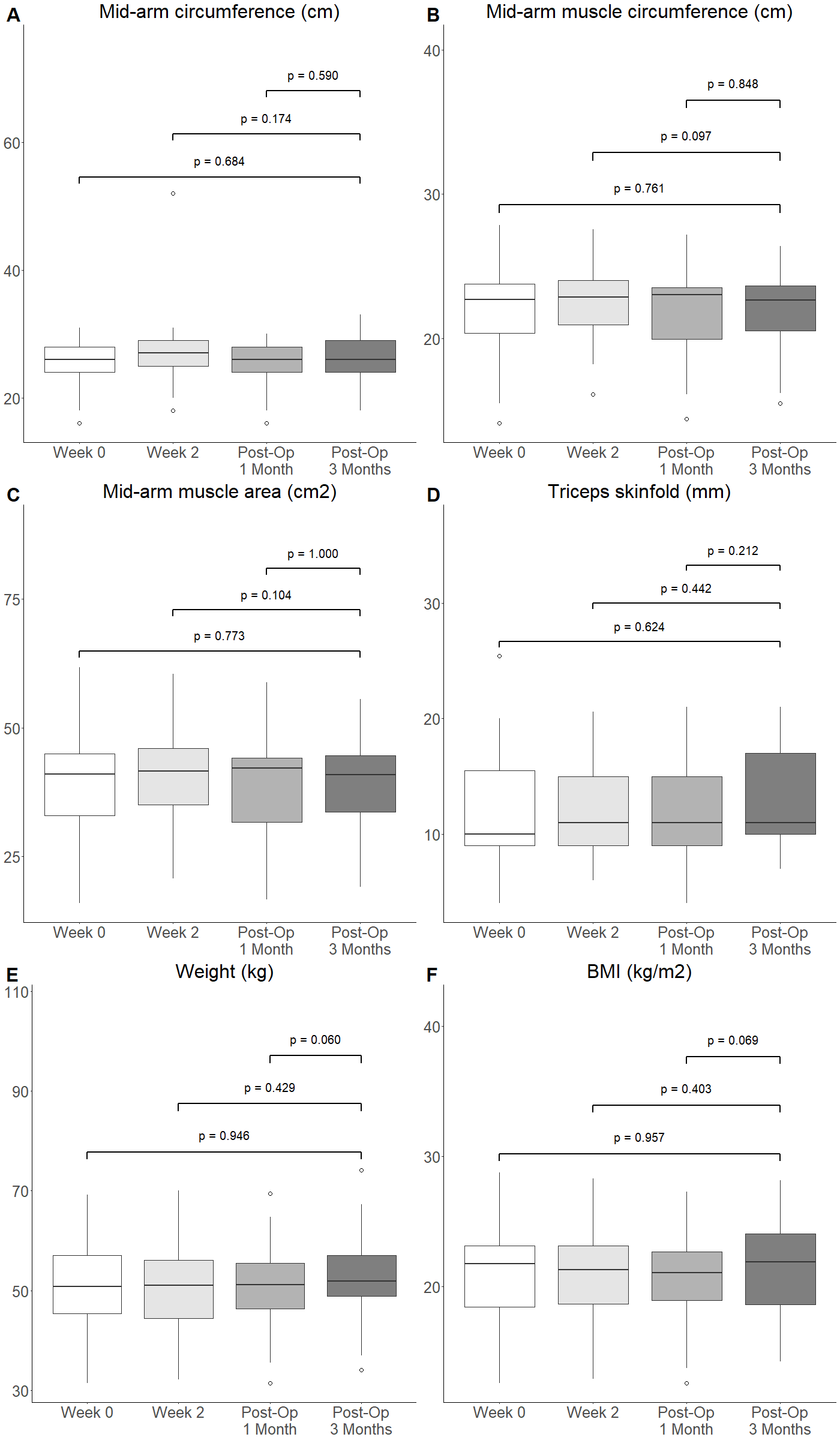

Supplement: Supplementary file 1 [file nutrients-18-00703-s001.zip › Supplementary Figure S4.png]

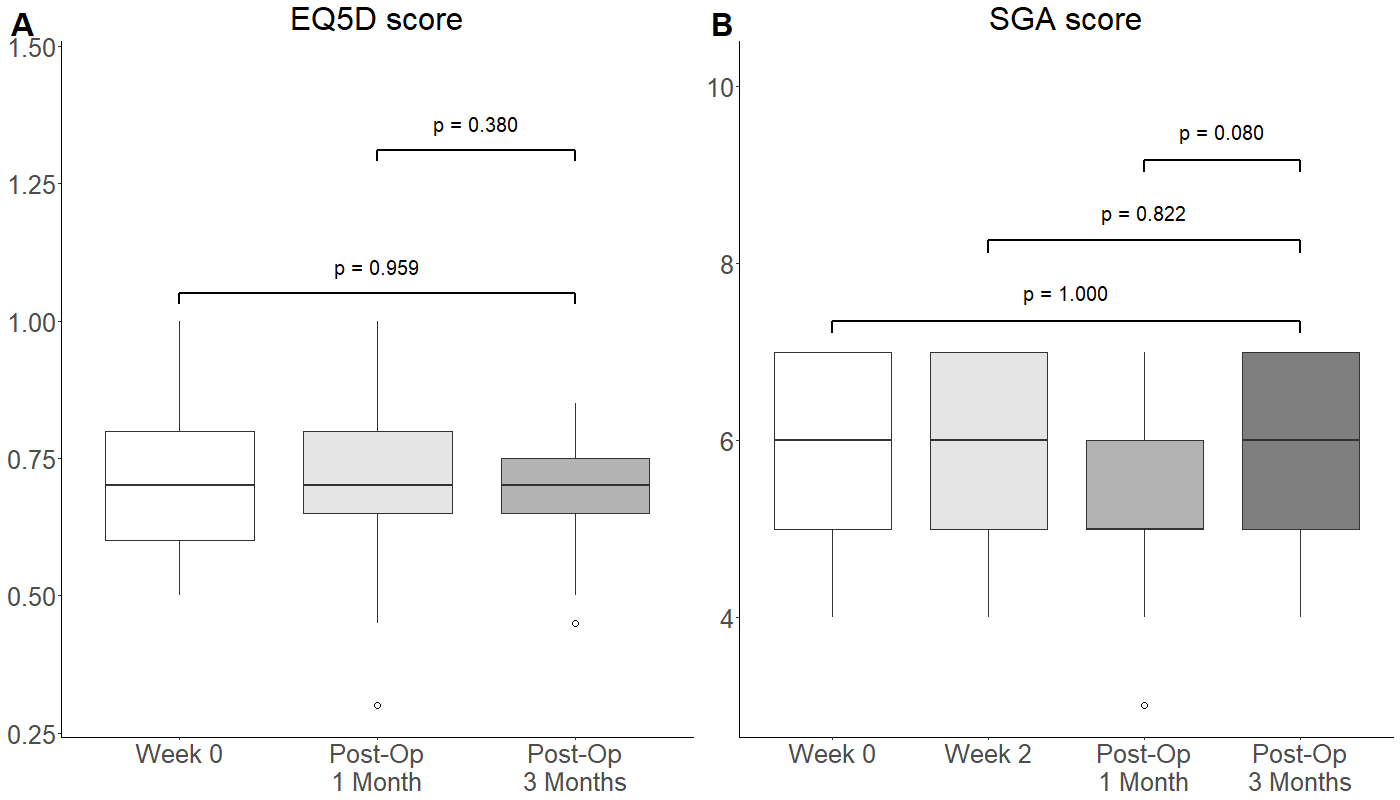

Supplement: Supplementary file 1 [file nutrients-18-00703-s001.zip › Supplementary Figure S5.png]

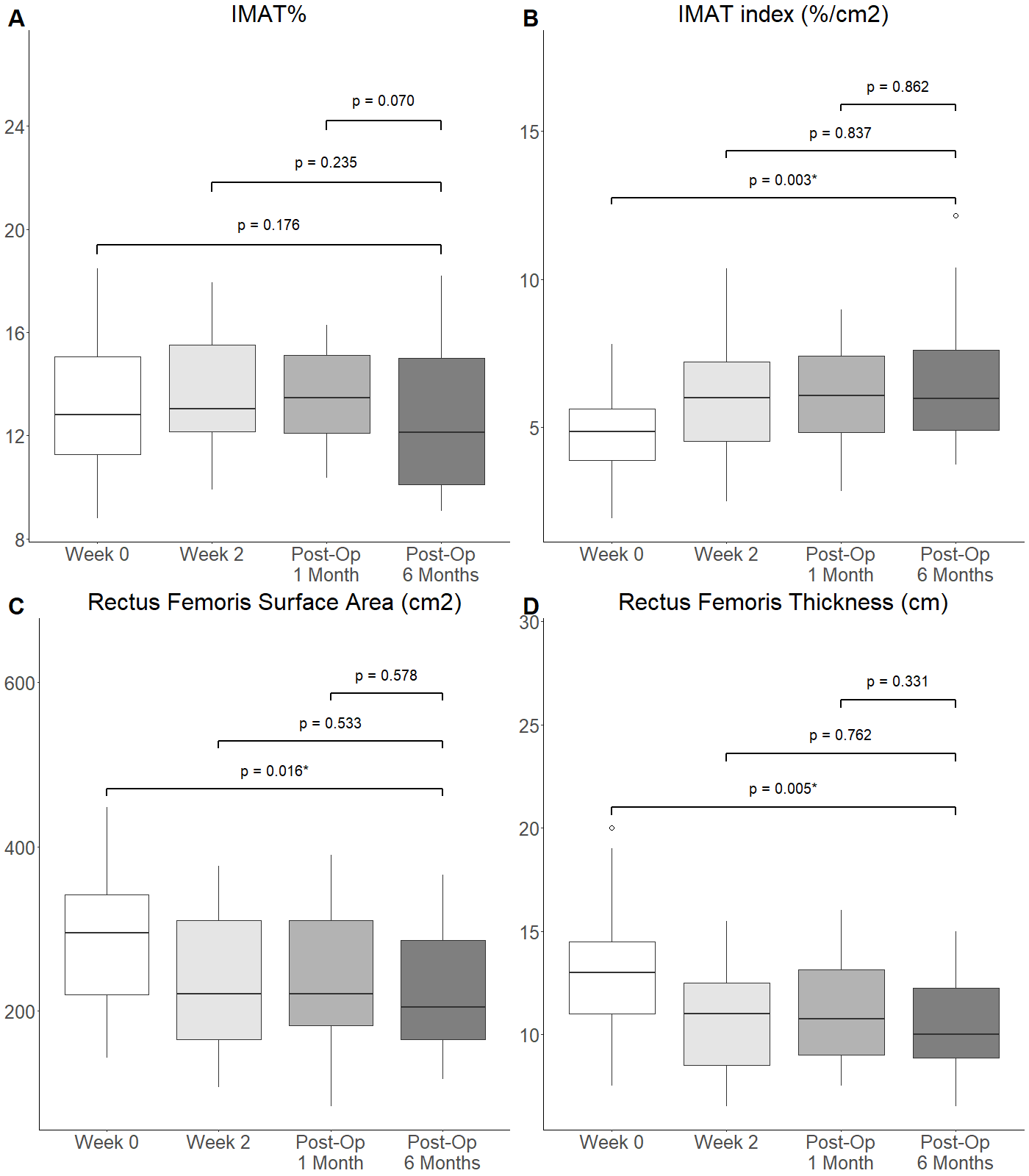

Supplement: Supplementary file 1 [file nutrients-18-00703-s001.zip › Supplementary Figure S6.png]

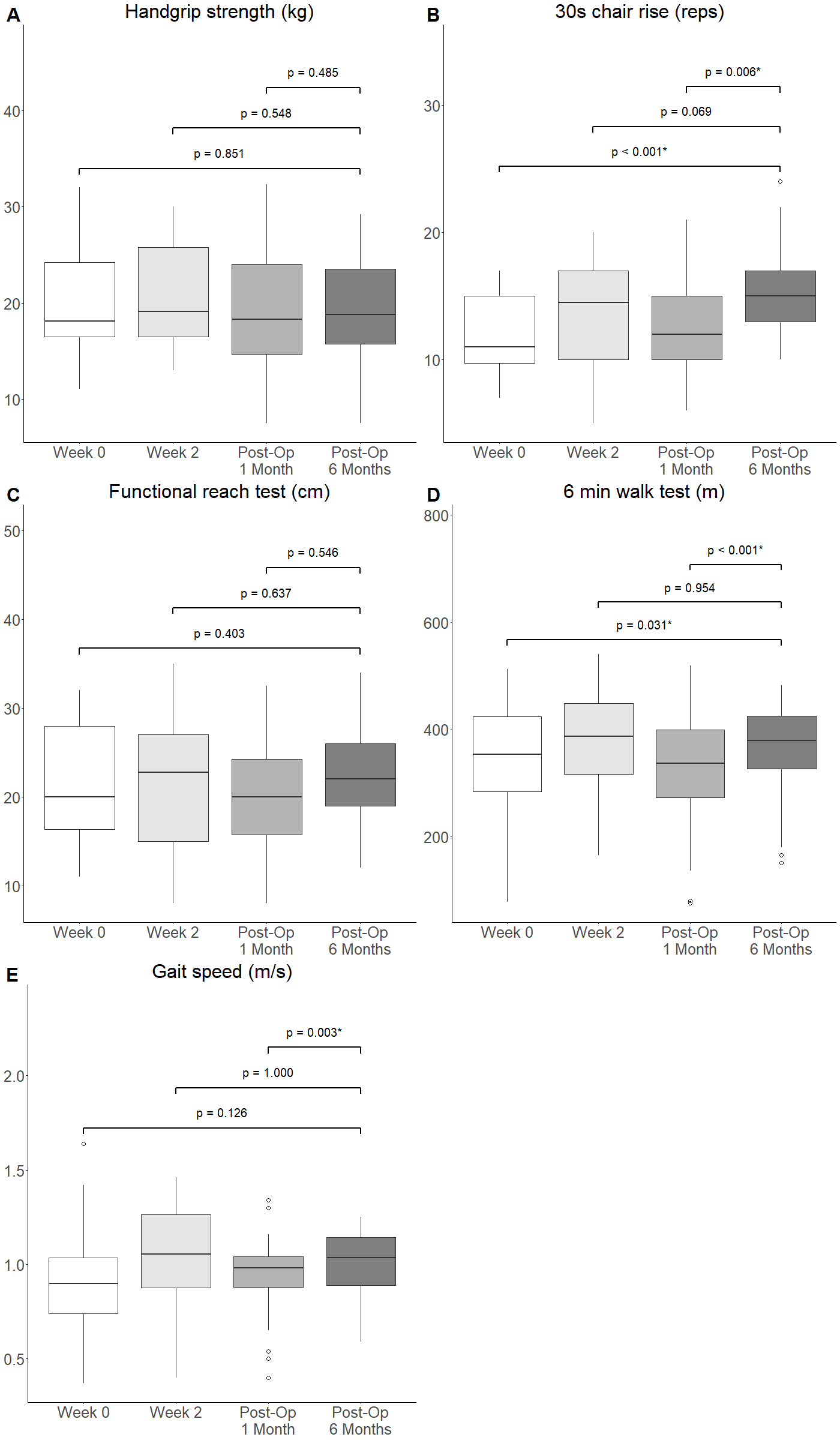

Supplement: Supplementary file 1 [file nutrients-18-00703-s001.zip › Supplementary Figure S7.png]

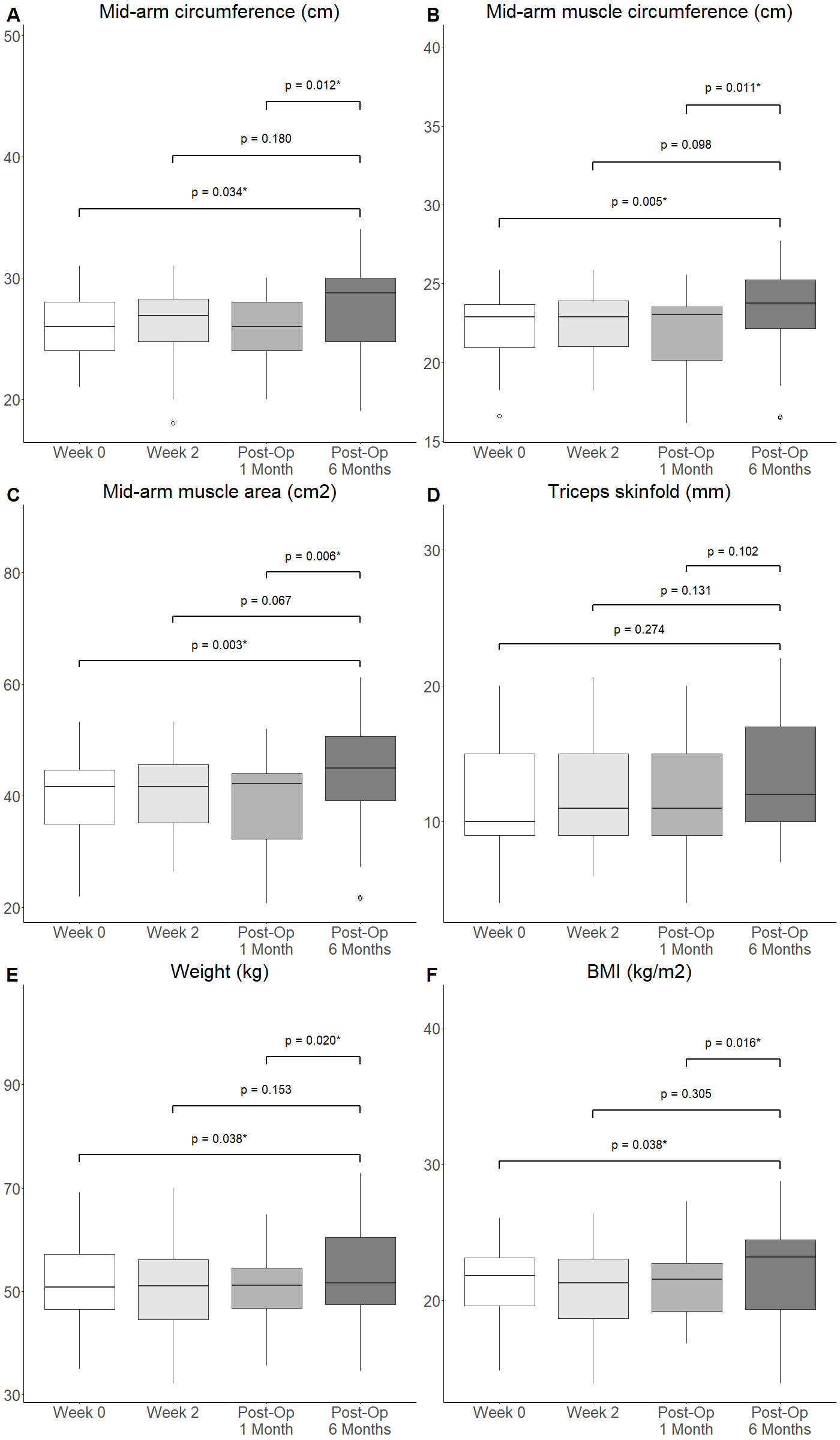

Supplement: Supplementary file 1 [file nutrients-18-00703-s001.zip › Supplementary Figure S8.png]

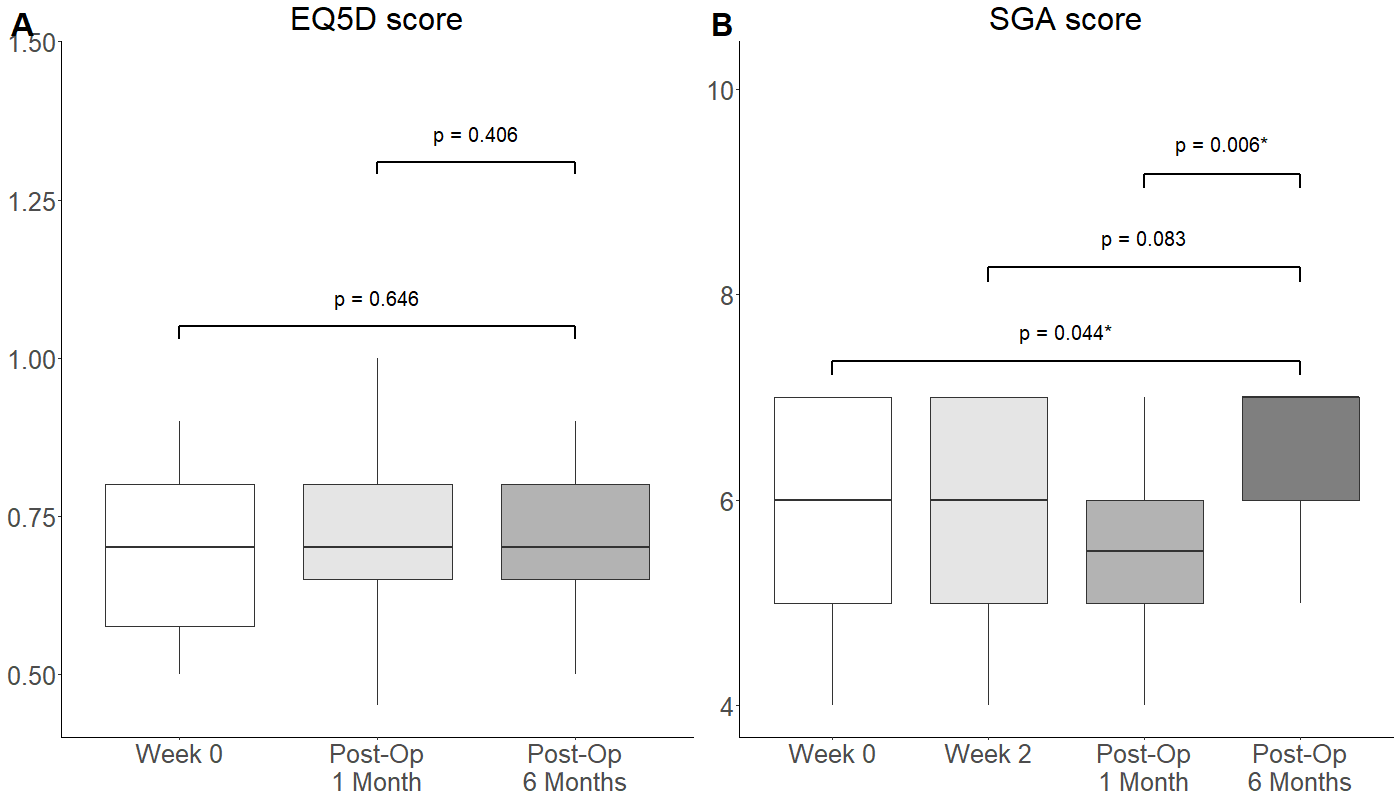

Supplement: Supplementary file 1 [file nutrients-18-00703-s001.zip › Supplementary Figure S9.png]
